# Using machine learning to distinguish between authentic and imitation Jackson Pollock poured paintings: A tile-driven approach to computer vision

**DOI:** 10.1371/journal.pone.0302962

**Published:** 2024-06-17

**Authors:** Julian H. Smith, Caleb Holt, Nickolaus H. Smith, Richard P. Taylor

**Affiliations:** 1 Cloudsmiths LLC, Eugene, OR, United States of America; 2 LightningHolt LLC, Eugene, OR, United States of America; 3 Fractals Research LLC, Eugene, OR, United States of America; 4 Physics Department, University of Oregon, Eugene, OR, United States of America; Industrial University of Ho Chi Minh City, VIET NAM

## Abstract

Jackson Pollock’s abstract poured paintings are celebrated for their striking aesthetic qualities. They are also among the most financially valued and imitated artworks, making them vulnerable to high-profile controversies involving Pollock-like paintings of unknown origin. Given the increased employment of artificial intelligence applications across society, we investigate whether established machine learning techniques can be adopted by the art world to help detect imitation Pollocks. The low number of images compared to typical artificial intelligence projects presents a potential limitation for art-related applications. To address this limitation, we develop a machine learning strategy involving a novel image ingestion method which decomposes the images into sets of multi-scaled tiles. Leveraging the power of transfer learning, this approach distinguishes between authentic and imitation poured artworks with an accuracy of 98.9%. The machine also uses the multi-scaled tiles to generate novel visual aids and interpretational parameters which together facilitate comparisons between the machine’s results and traditional investigations of Pollock’s artistic style.

## Introduction

In 1952, the Abstract Expressionist Jackson Pollock poured fluid paint onto a vast canvas rolled out across his studio floor and created his masterpiece, *Blue Poles*: *Number 11*, *1952* [1]. The painting represents the culmination of 10 years of developing his ‘pouring’ technique and the ‘all-over’ style that it generated. In contrast to conventional brush contact with the canvas surface, the constant stream of paint produced continuous trajectories that wove together into a uniform pattern that lacked conventional compositional values—no center of focus, no up or down, and no left or right.

As artistic recognition for his revolutionary style grew and the commercial value of his work soared, judgments of authenticity became increasingly crucial. A number of damaging controversies have plagued the Pollock world, fueled by painting prices that exceed $100M and by the growing number of fakes appearing on the art market [2,3]. If a poured painting of unknown origin is found today, how could we determine with reliability whether it is a masterpiece or a fake? In addition to the staggering financial consequences, rigorous methods are needed to protect the legacies of our most treasured artists.

Previous Pollock controversies were escalated by incorrect attributions made by well-respected Pollock experts, highlighting the challenge that the visual complexity of Pollock’s images presents for the human eye. This challenge forms the central scientific question of our study. If we replace the human observer with an artificial intelligence (*AI*) machine, what level of machine accuracy (*MA*) could be achieved when classifying the complexity of poured artworks into Pollock and non-Pollock categories? A *MA* close to 100% would suggest that artificial intelligence can distinguish Pollock’s artistic signatures more readily than some of the best Pollock scholars. Although the capability of *AI* machines to out-perform humans is not unusual—indeed, this ability fuels many current applications across society—it presents a unique dilemma for the art world. Pollock’s work was created for appreciation by humans and not machines. Perhaps the machine’s superior ability to distinguish between masterpieces and imitations represents *AI*’s version of art appreciation?

A high *MA* would also resolve a debate that has troubled the Pollock world from the moment he started to create his unusual patterns. Although many art theory essays have celebrated Pollock’s unique talent, the fact that Pollock scholars can sometimes fail to spot fakes fuels the public perception that his work is no more than an inevitable consequence of pouring paint—and that a lay person could readily match Pollock’s artistic achievements. Although the *AI* machine can’t judge aesthetic worth, a high *MA* value would provide objective and quantitative proof that Pollock’s work is a unique form of artistic expression.

In recent years, a variety of *AI* techniques have been applied to a growing number of other artists. An obvious approach for paintings featuring faces is to use *AI*-powered facial recognition methods. Focusing on the Renaissance master Raphael, this strategy identified a 97 percent similarity between the face of the Virgin Mary depicted in his confirmed painting *Sistine Madonna* and the face in the disputed work *de Brecy Tondo* [4]. In contrast to training on the ‘form’ of the painted images (e.g. faces, figures,etc), an alternative strategy is to focus on the tell-tale painting techniques used by artists to generate the images. An example study examined more than 80,000 individual brushstrokes by Picasso, Matisse, and Schiele and classified them with an accuracy of up to 90% [5]. Similarly, a team known as *Art Recognition* focused on artistic techniques such as brushwork and use of color, along with object placement within the canvas and other compositional characteristics. Their recent projects include confirming the authenticity of a van Gogh self-portrait and determining that an alleged painting by Max Perchstien is in fact by the infamous forger Wolfgang Beltrach [6]. Devoid of the illustrative content of traditional art and constructed from splatters rather than careful brushwork, what would be the optimal *AI* approach for Pollock’s work?

The authors formed an art-science collaboration called *Art Intelligence* to quantify *MA* performance as a way of gauging *AI’s* potential to ‘understand’ Pollock’s work. To promote the acceptance of this *AI* approach within the art world, we focus on well-established rather than novel machine learning models. Models will be more influential when resolving future Pollock controversies if they have a robust track record for addressing crucial applications across society. For the models considered in our study, these applications include analyzing faces at airports to maintain our national security and scanning medical images to ensure our health [7,8].

Accordingly, we train our machine to learn the visual characteristics of poured artworks using an artificial neural network called *ResNet* [9] and by exploiting an approach called Transfer Learning. *ResNet* employs ‘deep’ networks consisting of many layers and nodes (’neurons’) that process information hierarchically. By employing *ResNet’s* many hierarchical layers, the machine can detect a vast array of everyday visual signatures. We complement this machine architecture by reserving the last few layers of our neural network for discriminating between artworks by Pollock and those not by Pollock. We do this by showing the network the largest ever assembled digital collection of Pollock artworks, imitations of Pollock artworks, and a variety of other abstract artworks. We compare the performance of various versions of *Resnet* to other established machine models (including *AlexNet*, *DenseNet*, and *SqueezeNet)* and more recently developed Vision Transformers (including *Pyramid Vision Transformer*, *Swin Transformer*, and *Multi-Axis Vision Transformer*). Ultimately, we focus on *Resnet50* based on its superior *MA* and proven track record across a variety of applications [7,9,10].

Whereas the chosen model architecture is deliberately traditional, the novel aspects of our study focus on the input (image ingestion) and output (interpretational parameters) stages of the process. In terms of input into the machine, we emphasize that although our collection of 588 works is comprehensive it is nevertheless significantly smaller than data sets typically used for machine learning. For example, cosmologists apply *AI* to thousands of images of the night sky [11]. However, these same limits apply to human inspection of Pollock’s work. We therefore anticipate that *AI* will provide a valuable step forward in bringing state-of-the-art scientific techniques for spotting and quantifying patterns in complex data to the art world. The machine’s ability to perform these tasks can be expected to improve substantially if novel strategies can lift the restrictions presented by the limited image sets. How can the data input be boosted in order to achieve a high *MA* for Pollock’s work? This forms the second scientific question of our study.

To accommodate the small number of images, our machine learns about the artworks by dividing each work into an array of tiles. This image ingestion method exploits 2 key characteristics of Pollock’s all-over style. Firstly, because of the spatial uniformity of the all-over style, tiles at different locations are expected to have similar visual features to each other. Secondly, previous investigations examined the occurrence of fractal patterns [12] in the all-over style [13–34]. We therefore use many tile sizes at each location. Because of the scale invariance of fractals, these multi-scaled tiles are expected to share visual features. In effect, each of Pollock’s artworks features multiple ‘sub-Pollocks’ found at different locations and at different size scales—each displaying Pollock’s tell-tale signature. Our fractal tiling approach therefore aligns with the fractal character of the images and by doing so boosts the amount of art images that we can use during the training of our machine. In total, a data set of 97,275 Pollock tiles and 150,242 non-Pollock tiles is employed in our process.

By integrating this novel ingestion method into our current model, our machine distinguishes between Pollock poured paintings and artworks created by other artists with a *MA* of 98.9%. When the machine is presented with an artwork of unknown origin, it generates a Pollock Matching Factor (*PMF*) to quantify the degree to which the artwork captures the visual appearance of Pollock’s work using a scale from 0 to 1. Using our current model, we find that artworks with *PMF* values that reach or exceed the *PMF* ‘Threshold’ of 0.56 can be considered a close match to this visual appearance. This Threshold is set at the *PMF* that maximizes the *MA* for distinguishing between the Pollock and non-Pollock works. The machine gains confidence in the visual match to Pollocks as the *PMF* increases beyond the Threshold and similarly gains confidence in a miss-match as the *PMF* decreases below the Threshold.

Although the ‘black box’ quality of artificial neural networks highlights their immunity to human influence, this same quality limits insights into the visual signatures that the machine is employing. Our third scientific question therefore focuses on developing strategies to understand the machine’s output. In particular, can our *AI* approach provide a new ‘eye’ on Pollock’s all-over style and how it developed over a decade of refinement? As with the input strategy, the innovative step relies on our employment of multi-scaled tiles. We use these tiles to generate Pollock ‘Maps’ that examine the artistic signatures at different locations across the artwork and Pollock ‘Zoom-in charts’ that examine the signatures at different size scales. These visual aids are coupled with 4 parameters that allow us to quantify the key aspects of Pollock’s revolutionary style. These provide an interdisciplinary bridge between our new scientific approach and the wealth of ideas presented in traditional art theory studies of Pollock’s work. We show that fractality and spatial uniformity are important signatures used by our machine when identifying Pollocks. In particular, we show a strong correlation between *PMF* and the scale invariance of Pollock’s signatures, indicating that fractal detection is prominent in the machine’s decisions.

Given the prevalence of fractals in nature’s scenery (for example, trees, clouds, mountains, rivers, and coastlines are all shaped by fractals [12]), our machine provides an objective connection between Pollock’s poured paintings and nature. Frequently referred to as being natural in appearance, his complex patterns can be seen as a direct distillation of nature’s geometry onto his canvases, supporting the description of ‘Fractal Expressionism’ [35]. Although our parameters are developed for art interpretation of Pollocks, analogous approaches could readily be employed in other interdisciplinary studies of complex data aimed at establishing connections between traditional and novel interpretations of a subject. In particular, our novel tiling approach and its associated interpretive tools are well-suited for *AI* analysis of fractal patterns. Given the prevalence of fractals in nature, potential future fundamental and applied investigations include medical imaging of fractals in the body (e.g. tumors, neurons, veins, bronchial trees, bone fissures) [36], meteorology (e.g. clouds, rain, lightning, wind patterns) [12], geography (e.g. trees, plants, rivers, coastlines, mountains, mud cracks, rock textures) [12], and astronomy (e.g. dark matter distributions, star clusters, moon craters) [37–39]. Artificial physical systems could also benefit from our *AI* analysis. This includes investigations of the fractal distributions of transport routes [40].

We discuss our novel input and output approaches relative to other techniques to emphasize their distinct characteristics. Finally, we emphasize that our technique is not intended to be applied in isolation when used for attributing a poured work to Pollock. The results will be most useful for authenticity studies when coupled with other important information such as materials analysis, provenance, and connoisseurship. Notably, we expect our *AI* inspection to complement the visual inspections of Pollock experts in a unique marriage between computer and human vision. Inevitably, this marriage will be tested by the growing prevalence of *AI*-generated art. We show that our current *AI* technique can detect current *AI-*generated Pollock imitations. As techniques develop, it will be fascinating to see if the ability to detect Pollock imitations continues to outpace the ability to generate them. Perhaps future *AI* machines will become so proficient that they can not only replicate Pollocks but develop his style in new directions based on subtle career developments spotted by the machine. Within this context, we emphasize that our current study is simply the preliminary step to many future studies of abstract artworks.

## Background

**Fig 1:**
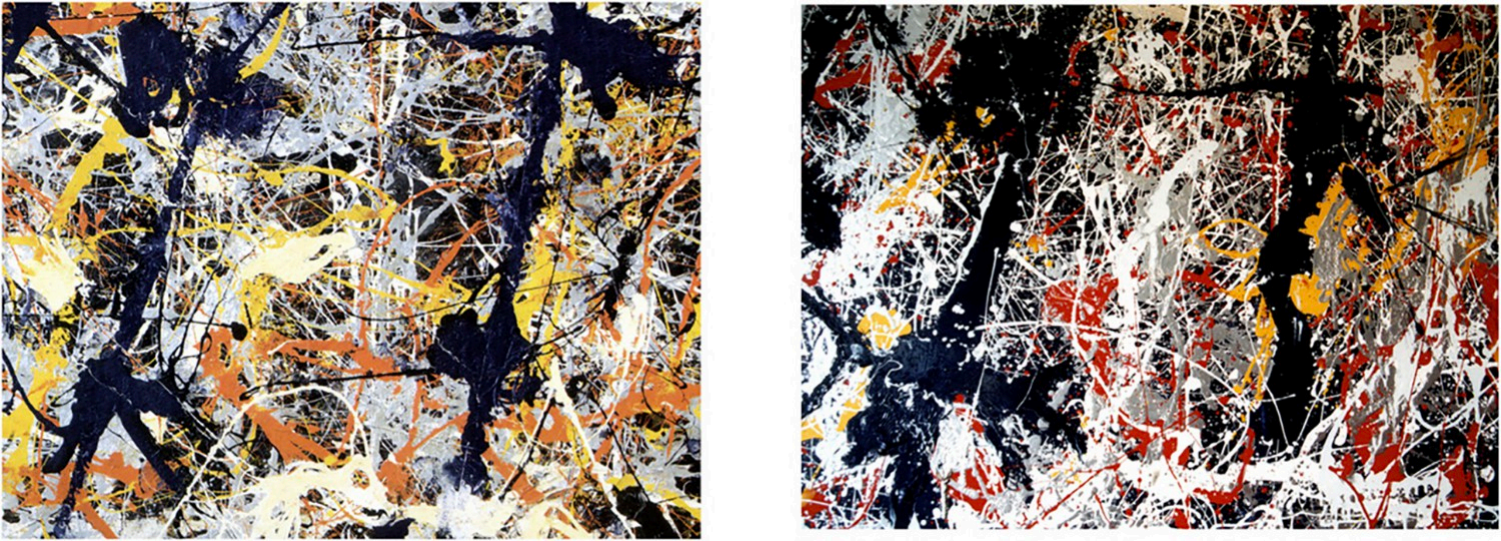
81 x 110cm sections of the real (left) and replica (right) *Blue Poles*: *Number 11*, *1952* (210 x 486.8cm, National Gallery of Australia).

Fig 1 highlights the challenge faced by authenticity studies by showing Pollock’s *Blue Poles* side by side with a known imitation. Investigations aimed at distinguishing real from imitation artworks need to probe beyond the superficial similarities displayed by these artworks. To do so, investigations typically integrate artistic and scientific approaches. From the sciences, a range of analysis techniques can be employed to determine the material composition and date of the paint, the canvas, and the frame. From the arts, provenance studies assess the painting’s history while connoisseurs conduct visual comparisons with established paintings from the artist’s catalog (in Pollock’s case, the *Catalogue Raissonne* [41]) to determine if the ‘hand’ of the artist is present.

For many artists, this combination of research tools yields powerful evidence for attributing paintings. Unfortunately, Pollock’s history adds substantial confusion to these approaches. Pollock is known to have bartered some paintings and so authentic paintings might exist for which the provenance is long lost. Pollock was also the subject of a film at his career peak. This inspired a wave of imitation poured paintings that date from Pollock’s era and are composed from similar materials. Because of these factors, visual inspection forms the central cornerstone of Pollock investigations. However, when faced with the staggering commercial implications coupled with the pressures of international media attention, subjective judgments have become increasingly difficult to obtain and defend against potential litigation. This creates an art world vulnerable to the acceptance of fakes and to the denial of long-lost treasures.

In response, one of this paper’s authors (Taylor) pioneered the use of computer analysis to supplement the visual experts’ inspection with quantitative and objective pattern detection techniques [13]. Based on frequent references to the “organic” and “natural” appearance of Pollock’s all-over style, along with Pollock’s declaration that “my concerns are with the rhythms of nature” [1], this computer analysis focused on fractals. The analysis quantified a key visual element of Pollock’s work—the fractal complexity generated by the patterns that repeat at increasingly fine size scales [12]. Hints of their telltale pattern repetition had already been noticed in Pollock’s era by art critics, journalists, and Pollock himself: “knit together of a complicity of identical and similar elements” (Clement Greenberg) [1], “The large in Pollock is an accumulation of the small “(T.J. Clark) [1], “patterns all roughly similar in character … over the surface of the picture” (William Rubin) [1], “Pollock is as strong from a distance as he is close to” (Alfred Frankenstein) [1], and “my paintings didn’t have any beginning or end” (Pollock) [1].

Commencing with Taylor’s initial publication in 1999 [13], 13 groups have since used various forms of fractal analysis to explore Pollock’s artistic signature [13–34]. In each case, computers were used to investigate similarities in the statistical characteristics of the painted patterns occurring across different size scales. In particular, the fractal dimensions (*D*) of Pollock’s poured patterns have been employed to distinguish his artistic signatures from those of his imitators [17]. Two groups have also used fractal techniques to create computer-generated imitation Pollocks [26,33]. Subsequent neuroscience experiments highlighted the aesthetic impact of fractals [42,43]. In particular, demonstrations of the shared visual qualities of nature’s and Pollock’s fractals inspired the Fractal Expressionism art movement [35].

The scholarly investigation of Pollock’s fractals informed a high-profile Pollock authentication investigation in 2005 when the Pollock-Krasner Foundation collaborated with Taylor to determine the origin of the ‘Matter Collection [2,20]. Fractal analysis was applied to 6 of 32 newly discovered poured paintings. These were found to differ in fractal characteristics [17] from 14 established Pollocks, consistent with subsequent pigment analysis showing that some of the paints dated from after Pollock’s death. In another investigation of the ‘Knoedler Collection’ [3] by the International Foundation for Art Research, fractal analysis again highlighted fractal differences between one of the paintings and established Pollocks. This was consistent with the later discovery that the paintings were created by a contemporary artist. Although fractals have therefore played a useful role in authenticity studies, Taylor emphasized that they are one of many visual characteristics needed to capture the rich experience of viewing Pollock’s paintings [17]. Identifying a more comprehensive set of Pollock’s visual characteristics would lend power to the process of separating his masterpieces from their imitations.

Could a computer train itself to identify and learn from a set of pattern characteristics in an artwork? Although asking a computer to make decisions about $100M paintings might at first seem to place too much trust in a novel technique, *AI* is already being employed to perform critical functions for society. In parallel, *AI* is experiencing a growing role in generating, classifying, and examining the authenticity of diverse works across the arts (for a recent review, see [44]). Prominent teams using *AI* for authenticity studies of paintings include *Artrendex* and *Art Recognition* [6,45]. Table 1 presents some further examples presented in chronological order [46–52].

**Table 1:** A selection of *AI* techniques listed in chronological order.

Reference	Approach	Data Set	Measure	Result
Qi et al– 2013 [46]	Wavelet Hidden Markov Tree	Impressionist/Post-Impressionist	Attribution Accuracy	85–88%
Shamir– 2015 [47]	Weighted Nearest Neighbors	Jackson Pollock	Painting Accuracy	93%
Van Noord et al– 2015 [48]	AlexNet (*CNN*)	Rijksmuseum Dataset	Mean classification accuracy	78%
Liu et al—2016	Geometric Tight Frame	Vincent van Gogh	Painting Accuracy	87–89%
Van Noord et al– 2017 [50]	Multi-scale *CNN*	Rijksmuseum Dataset	Mean class accuracy	82%
Dobbs and Ras– 2022 [51]	ResNet101 (*CNN*)	RIjksmuseum Datasest	Mean class accuracy	91%
Schaerf et al– 2023 [52]	EfficientNetB5 (*CNN*)	Vincent van Gogh	Painting Accuracy	96%
Schaerf et al -2023 [52]	Swin-Tiny (*ViT*)	Vincent van Gogh	Painting Accuracy	87–88%

In the early 2010s, attempts at art authentication such as those of Qi et al [46] relied on more traditional computer vision techniques coupled with Fisher Information scores to categorize artworks. Because their research focused on the brushstrokes of Impressionist and Post-Impressionist art, their approach might not be expected to transfer well to the task of identifying Pollock’s poured paintings. Nonetheless, in 2015 Shamir employed a similar technique to discriminate between Pollock forgeries and Pollock originals using a Fisher Information score after extracting a set of 3000 visual features [47]. Despite the small data set of 26 Pollock poured works, his machine learning technique achieved a *MA* of 93%. Interestingly, we note that Shamir found that the fractal parameters in the set were the dominant distinguishing tools used by the machine.

By 2015 a crucial shift was underway in the computer vision world in the form of the deep learning capabilities of artificial neural networks. Convolutional neural networks (*CNNs*) were becoming popular and, significantly, the paper detailing *Resnet* was published at this time [9]. *CNNs* make use of a limited set of “filters” which are then convolved mathematically across an image. By reducing the input layer to a small number of filters, *CNNs* perform well at approximating the visual characteristics of animals, and out-perform traditional neural networks when it comes to feature extraction [53]. Accordingly, in 2015 van Noord et al applied a *CNN* (*AlexNet*) to the Rijksmuseum data set and demonstrated that *CNNs* could be effective at art authentication. Subsequently, van Noord et al expanded their approach and achieved a higher classification accuracy on the same data set by adopting a multi-scale *CNN* [50]. Dobbs and Ras have achieved the highest *MA* so far on the Rijksmuseum data set by using *Resnet101*. Further improvements utilizing *EfficientNetB5* were developed by Scharf et al (2023) who were able to classify works by van Gogh with a *MA* of 96%.

Another form of deep learning—Vision Transformers (*ViTs*)—drives the latest trends in computer vision. *ViTs* ‘tokenize’ images in order to digest their information. This image tokenization is similar to how the transformers in large-language models tokenize words and sentences to digest text. In both cases, transformers decompose the data sets into smaller chunks and then extract information from the chunks [54]. Last year, Schaerf et al demonstrated the effectiveness of *ViTs* for identifying art-works by Vincent van Gogh.

Although Pollock’s abstract artworks have been the focus of high profile authenticity controversies involving staggering financial and artistic consequences, they have not yet benefited from an *AI* deep learning analysis. Given *ResNet*’s established history in art classification and other applications, we focus our model comparisons on *ResNet* architectures along with a number of other *CNNs*. We also include several *ViTs* because of their emerging status in *AI* applications.

Considering our novel image ingestion approach, we note that our integration of tiling into the *ResNet* model is similar in some ways to the tokenizing approach utilized by *ViTs*. However, our tiling strategy differs in one key aspect: when our technique decomposes the images into tiles, we deliberately discard their relative positions while *ViTs* retain this information. Our tiling strategy is based on the fractal composition of Pollock’s all-over style in which each tile serves as an *independent* Pollock image. As such, our study uses deep learning networks in a manner consistent with the previous scientific studies indicating that fractal parameters are useful descriptions of Pollock’s complex patterns. Training our neural network on an array of multi-scaled tiles is therefore an intriguing solution to boosting image numbers while aligning our investigations with previous quantitative approaches to Pollock’s artistic signatures. Our unique tiling approach, and its alignment with fractal patterning, also establishes the novelty of our interpretative Zoom-in Charts and Maps. For example, whereas other *AI* approaches identify the spatial locations of interesting features (e.g., unusual brushstrokes in paintings [6]), our Maps instead compare the visual signatures of well-defined regions of the canvas at different locations and size scales.

## Methods

### Image sets

The images of the 588 artworks used in our study were acquired in collaboration with The Pollock-Krasner Foundation, The Pollock-Krasner Study Center, The International Foundation for Art Research, and Francis V. O’Connor (chief Pollock connoisseur and co-author of the *Catalogue Raissonne)*. The collection and analysis method of all images complies with the terms and conditions for the sources of the data. The S1 Table provides a comprehensive list of the image sets. The image sets feature 2 overall categories of artwork—those established as being created by Pollock and those established to be by other artists.

#### Pollock poured artwork

This category features all 189 Pollock artworks that satisfy our definition of poured works and that have known images. Pollock used a variety of painting techniques across his career [1]. Our definition of pouring is broad and is based on artworks that feature patterns generated by fluid liquid poured onto the artwork’s surface. To ensure our technique is robust to variations in image quality and color variations, we use multiple images of each painting when possible. More specifically, we employ 2 collections: one set features color images typically with high image resolution (scanned at 1200 px/inch from a variety of high quality art books [55–67]) and the other set features grayscale images with lower but still good image resolution (scanned at 600 px/inch from the Pollock’s *Catalogue Raissonne* [41]). Using this approach, 118 of the 189 Pollock artworks feature images from both sets.

#### Non-Pollock art works

This second category comprises 2 groups of artwork. The first group features a diverse range of 284 poured works created by other artists. These include artworks generated specifically for this authenticity project (e.g. 32 adult and 18 children’s paintings created under the controlled conditions of ‘Dripfest’ events [17,31], and paintings generated by mechanical devices such as the Pollockizer [35]), 100 commercially-available poured artworks, poured artworks by well-known artists (e.g. by Michael Baldwin, Max Ernst, Sam Francis, Arshile Gorky, Hans Hofmann, Henri Michaux, Norman Rockwell, Niki de Saint Phalle, etc) and established poured imitations (e.g from the Knoedler Collection, the Matter Collection, by Mike Bidlo, Ed Harris, and Francis O’Connor). To add robustness, the non-Pollock category also features a diverse group of 115 abstract ‘non-poured’ abstract works by famous artists (e.g. by George Braque, Jasper Johns, Wassily Kandinski, Paul Klee, Willem De Kooning, Joan Miro, Clyfford Still, etc) [68]. Only 1 image of each artwork is used in the non-Pollock category. The majority of the images are scanned at 1200 px/inch from a variety of high quality art books, prints, and transparencies. Photographed works (Dripfest works, *One* by the Pollockizer, *Untitled* by the Wind Machine, and *Dummy* by Richard Taylor) use high resolution settings (200–400 px/inch). Images of the commercial poured paintings are downloaded from the internet (72 px/inch).

### Image pre-processing

Prior to training, images undergo a refined cropping process that employs a semi-automatic technique. Selecting the 4 corners of the painting manually, we then determine the minimum rectangle that encapsulates all of these points. We crop the image to this minimum rectangle. We tile each cropped image by covering the image with an array of identical squares and we then crop these to create a set of square sub-canvases. Based on the fractal model, we repeat this tiling process for various tile sizes, starting from squares with 10cm side lengths (the smallest tile width is set at 10 cm to avoid image resolution effects) and increasing in 5cm increments up to the maximum square size allowable for the painting. We center the tiling process on the image. As an example, for an image of size 265cm x 265cm we start the tiling process 2.5cm in from the left edge and 2.5cm down from the top edge. We continue until we reach the last tile, which is 2.5cm in from the right edge and 2.5cm up from the bottom edge. Once completed, we re-size each tile to a standardized 256 x 256 pixels and feed them into the neural network for classification. To illustrate this process, consider *Blue Poles*. We tile its 212.1cm × 488.9cm canvas with squares with side lengths ranging from 10cm up to 210cm, resulting in 2,387 tiles. Notably, our approach refrains from introducing any additional augmentations to the images beyond this pre-processing step.

### Data partitioning

Machine learning typically requires a set of images to train the machine (the ‘training set’) and a second set to iteratively evaluate the machine’s performance (the ‘validation set’). Traditionally, the validation set is used to evaluate machine models featuring different hyperparameters. In addition, we reserve a ‘hold out’ set of images that ensures that the iterative process of evaluating models with different hyper-parameters is not prone to any unseen biases. In other words, we ensure that the model will generalize to images that it has never seen. This is especially important for our circumstances whereby the set of known images of Pollock’s artworks is not anticipated to grow significantly. The hold out set differs from the validation set in that it is never used to make decisions for improving or tuning our model. We evaluate the performance of the different models using only the validation set. Once we decide on the final model, we then test it on the hold out set. We refer to the combination of the validation and hold out sets as the ‘inference set’.

Out of our 189 Pollock poured works, 142 (75.1%) are selected for the training set, 33 (17.5%) for the validation set, and 14 (7.4%) for the holdout set. For the Pollock images, the holdout set is randomly selected from the 189 paintings with the exception of deliberately including *Blue Poles* (as one of his most iconic images) and at least 1 painting from each year that he created a poured painting (i.e. 1943 and 1946–1955). Out of our 399 non-Pollock works, 304 (76.2%) are selected for the training set, 72 (18.0%) for the validation set, and 23 (5.8%) for the holdout set.

Importantly, all of the tiles extracted from an image in our training set are used during the model training process. None of the tiles extracted from an image in our training set are used during model validation or when testing the model using the holdout set. The same is true for both the holdout and validation images. In total, a dataset of 97,275 Pollock tiles and 150,242 non-Pollock tiles are collected. Table 2 summarizes the relative numbers of images dedicated to each role.

**Table 2:** Data partitioning of Pollock and non-Pollocks according to the number of artworks, the number of images of these artworks, and the number of tiles in these images.

Data Partitioning (artworks)			
	Train	Validation	Hold Out
Pollock	142 (75.1%)	33 (17.5%)	14 (7.4%)
non-Pollock	304 (76.2%)	72 (18.0%)	23 (5.8%)
Data Partitioning (images of the art works)			

	Train	Validation	Hold Out
Pollock	246 (75.7%)	51 (15.7%)	28 (8.6%)
non-Pollock	307(76.4%)	72 (17.9%)	23 (5.7%)
Data Partitioning (Tiles)			

	Train	Validation	Hold Out
Pollock	72,609 (74.6%)	12,983 (13.3%)	11,683 (12.0%)
non-Pollock	111,464 (74.2%)	31,173 (20.7%)	7,605 (5.1%)

### Training

The machine uses the tiles described above to learn about the visual features of the artworks. The machine compares all of the tiles within the established Pollocks to the tiles within works known to be created by other artists. During this comparison, the machine identifies a collection of visual features that are useful for distinguishing between the Pollock and non-Pollock artworks. Based on the features present in a tile, it then assigns a value from 0 and 1 to the tile, which we call the Pollock Signature. A Pollock Signature of 0 indicates that the tile does not have the distinguishing visual features of a Pollock work, while a value of 1 indicates that it does. Values closer to 1 indicate increasing levels of confidence from the machine that the artwork contains the distinguishing visual features of a Pollock. All tiles are considered equally in training.

**Fig 2:**
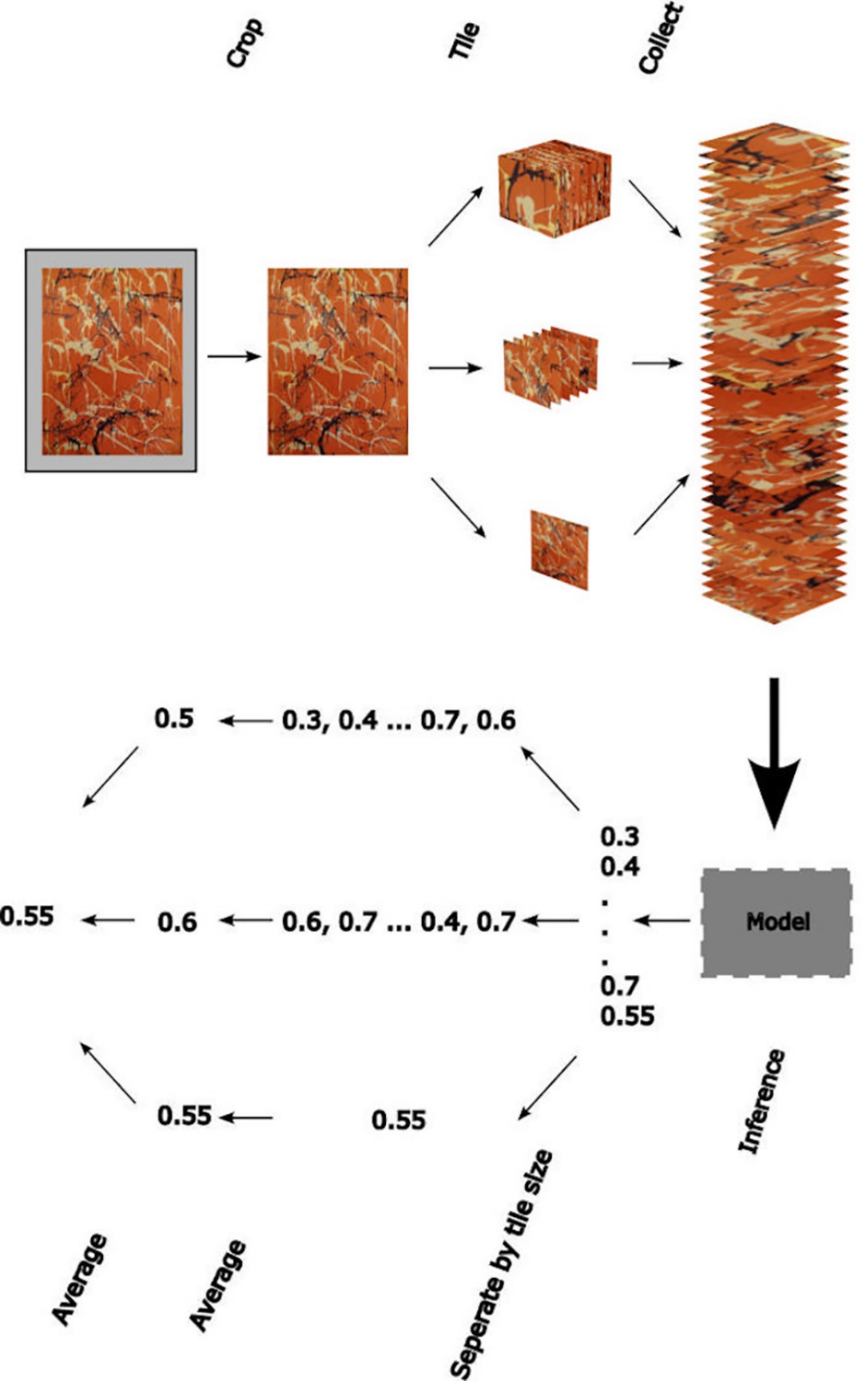
A flow diagram of the image pre-processing, data partitioning, and machine learning. The image is cropped and then tiled at multiple size scales (3 of the 22 tile scales are shown). The machine trains on the full set of tiles. During inference, the machine assigns a Pollock Signature to each of the tiles, which are then grouped by size and an average Pollock Signature is assigned. Finally, the average Pollock Signature is averaged across tile sizes to calculate the *PMF*. The intermediate numbers shown in the diagram are Pollock Signatures of the tiles and the final number is the *PMF*.

The machine then averages the Pollock Signature across all of the tiles (i.e. across different locations and different sizes) to generate a signature for the whole artwork called the Pollock Matching Factor (*PMF*). The *PMF* value lies on a scale from 0 to 1. A *PMF* of 1 indicates that every tile in the work displays a Pollock Signature of 1. Similarly, a *PMF* of 0 indicates that all tiles in the work have a Pollock Signature of 0. Increasing values above 0 indicate that more and more of the artwork’s tiles have a high (~1) Pollock Signature. A data flow diagram of the full training process is shown in Fig 2.

### Data quantification and visualization

#### The Pollock Dial

Because the *PMF* values vary from 0 to 1, we can visualize this information on a dial to easily compare *PMF*s of different artworks. The Pollock Dial plots the *PMF* of artworks using angular position with the *PMF* increasing in the clockwise direction. The Dial focuses on images from the inference set. Using the images on the Pollock Dial, we identify a *PMF* ‘Threshold’—artworks that reach or exceed the *PMF* ‘Threshold’ can be considered a close match to the visual appearance of Pollock’s works. This Threshold is calculated by determining the *PMF* that maximizes the *MA* when distinguishing between the collections of Pollock and non-Pollock works.

#### The Zoom-in charts and scaling parameters

The Zoom-In Charts examine how the Pollock Signatures vary with tile size. To generate the Charts, the machine groups tiles by their size and calculates the average Pollock Signature for each tile size. This average value is then plotted as a function of tile size using a bar chart. The Chart also displays the *PMF* of the whole artwork (which corresponds to the average across the tile sizes of all of their average Pollock Signatures). Bars with Pollock Signatures below the PMF Threshold are colored red while those above are green.

As discussed in the Introduction, previous research employed computers to perform a fractal analysis of Pollock’s poured paintings. For fractal artworks, the statistical qualities of the painted patterns repeat at increasingly fine size scales. To be consistent with fractals, we expect the Pollock Signatures to similarly repeat at different tile sizes. We introduce Scale Invariance (*SI*) to quantify the variation in the Pollock Signature with tile size. This is based on the root-mean-square variation across all tile sizes. Its range is normalized using the function *f(y)* = 1 - (2*y). Adopting this normalization, *SI* = 1 corresponds to no variation in Pollock Signature as we zoom in and *SI* = 0 corresponds to the maximum possible variation. We also introduce Magnification (*M*) to quantify the extent of the zoom-in, with *M* representing the ratio of the sizes of the largest and smallest tile widths.

#### The signature maps and spatial parameters

Whereas the Pollock Zoom-Ins examine the variation in Pollock Signatures with size scale, the Signature Maps examine how the Pollock Signature varies for different locations across the artwork. We generate a Map for each painting in the inference set. At each location, the Map averages the Pollock Signature of the various sized tiles at that location. In other words, at each pixel location we identify all of the multi-scaled tiles that contain this pixel. The Pollock Signatures of these tiles are then averaged. This value is plotted at the pixel location using color coding (red for values below the *PMF* Threshold and green for values above). During this process, we slide the arrays of tiles across the image and calculate the average of the Pollock Signatures across the various slide positions. This sliding technique generates a smooth map by eliminating any discontinuities caused by crossing the boundaries of tiles with large Signature differences. The completed Map allows us to identify regions of the painting that deviate away from Pollock’s style. We emphasize that, although related to the work’s *PMF*, these Map Signatures use a different averaging technique to the one used to generate the *PMF*.

We introduce 2 parameters to quantify spatial variations in the Map. Uniformity (*U*) quantifies the variation in the Pollock Signature across different locations. This is based on the root-mean-square variations of the Pollock Signatures in the width and height directions. The range of the variation in each direction is then normalized using the function *f(y)* = 1 - (2*y). Adopting this normalization, *U* = 1 corresponds to no variation and *U* = 0 corresponds to the maximum possible variation. The *U* value for the painting corresponds to the mean of the *U* values for the 2 directions. Coverage (*C*) employs a pixel count to quantify the relative sizes of the red and green coverage in the Map, with *C* = 0 corresponding to all locations being red and *C* = 1 corresponding to all regions being green.

#### Fractal analysis: Differential box counting and shift differential box counting

Some previous forms of fractal analysis of Pollock’s poured works extracted layers with different colors from the painting and then analyzed the pattern scaling properties of each of these layers separately [17]. Other approaches investigated the scaling properties of the ‘whole’ painting (ie. the combination of all of the layers in the painting). This is usually done by examining the luminance variations of the patterns. Because we train the machine on the ‘whole’ pattern, we adopt the luminance approach and apply Differential Box Counting (*DBC*) and Shift Differential Box Counting (*Shift-DBC*) algorithms to calculate the works’ fractal dimension *D*. These techniques are commonly used in the study of natural and synthetic patterns with fractal-like characteristics. They allow researchers to quantify and classify the structure of images from various disciplines, including geology, biology, and computer vision [69].

For the *DBC* technique, a square crop of the grayscale image of the artwork is converted into a 3D representation by plotting the pixel brightness in the height direction (normalized to the painting’s physical dimensions). This 3D space is covered with an array of identical ‘boxes’, each with a side-length *L*. At each location in the square crop, we then calculate the number of boxes that fit into the difference between the minimum and maximum grayscale values in the height direction. *NB* is the sum of these box counts across all of the locations. This calculation is repeated for arrays of boxes with different *L* values. A scaling plot is then generated which plots *NB* vs *L* on log-log scales. Fractal behavior is characterized by the power law relationship, *NB* ~ *L^−D^*, and *D* can therefore be extracted from the slope of the linear part of the scaling plot [69]. To convert this dimension in 3D space to the painting’s physical 2D space, a value of 1 is subtracted from the *D* value obtained from the scaling plot.

The Shift-DBC technique is an extension of DBC technique and introduces box shifting. Instead of placing boxes in a fixed grid, Shift-DBC shifts the box positions, allowing a more accurate estimation of *D*. The *D* value obtained from these 2 methods provides insight into how the image’s features repeat at different scales to generate the overall fractal pattern. In particular, because higher *D* values correspond to steeper slopes in the scaling graph, high-*D* images generate larger *NB* values at smaller scales than corresponding lower-*D* images. In this way, *D* quantifies the relative contributions of the fine and coarse patterns in the fractal image. It therefore serves to quantify the visual complexity of fractal patterns such as those found in Pollock’s works. *D* lies on a scale between 1 and 2 with simple, sparse fractals lying closer to 1 and rich, intricate fractals lying closer to 2.

### Model selection and performance

We train our machine using the model architecture *ResNet*, which is an abbreviation for *Residual Network*. This artificial neural network was introduced by researchers at Microsoft Research in 2015 and soon after won a general image classification challenge [9]. It is recognized as a powerful tool used frequently in computer vision tasks, particularly for recognizing objects in images. Artificial neural networks such as *ResNet* consist of many hierarchical layers analogous to those used by our brain when processing images. By employing *ResNet*, the machine can detect a vast array of everyday visual signatures. We complement this model architecture by reserving the last few layers of our neural network for discriminating specifically between artworks by Pollock and those not by Pollock. We do this by showing the network our digital collection of Pollock artworks, imitations of Pollock art works, and a variety of other abstract artworks. In this way, our machine transcends from the equivalent of an art novice to a Pollock expert.

More specifically, *ResNet*’s innovation over other neural networks lies in its introduction of ‘skip connections’ that help the network to learn and optimize more effectively [10]. Transfer Learning (*LT*) leverages knowledge gained from one task to improve performance for a different but related task. Instead of starting from scratch, *TL* uses a pre-trained model that has already learned basic features from a larger and more varied data set. We use *TL* to adapt *ResNet* from its task of classifying images featured in the *Imagenet* data set to our specific Pollock-related task [70]. *Imagenet-1K* provides images of a variety of objects, ranging from beagles to violins [71]. This pre-training saves time and computational resources, exposes the machine to a rich set of visual features, and improves the machine’s ability to generalize beyond a specific type of image. The initial layers of the pre-trained model are used as a foundation. The later layers are adjusted to fit our Pollock-detection task. For our project, we utilize *fastai* [72] and *timm* [73] model libraries for neural network architectures.

A list of hyperparameters that we employ during model development can be found in the S2 Table. *MA* is a crucial parameter for assessing model performance. Another criterion is minimizing the *PMF* difference between images of the same painting from different sources. The following hyper-parameters are also important to consider: learning rate, batch size, and number of epochs. Learning rate determines how fast a model learns from the data. A higher rate produces faster learning but runs the risk of overshooting without settling on a good solution, while a lower rate produces slower learning but benefits from being more precise. The batch rate is the number of tiles used in each iteration of training. The number of epochs is the number of times the entire training data set is processed by the model. We choose a learning rate of 10^−3^, a batch size of 64, and 1 epoch. We note that the high accuracy and performance is not increased by considering different epochs or batch sizes (see S1 Fig).

**Fig 3:**
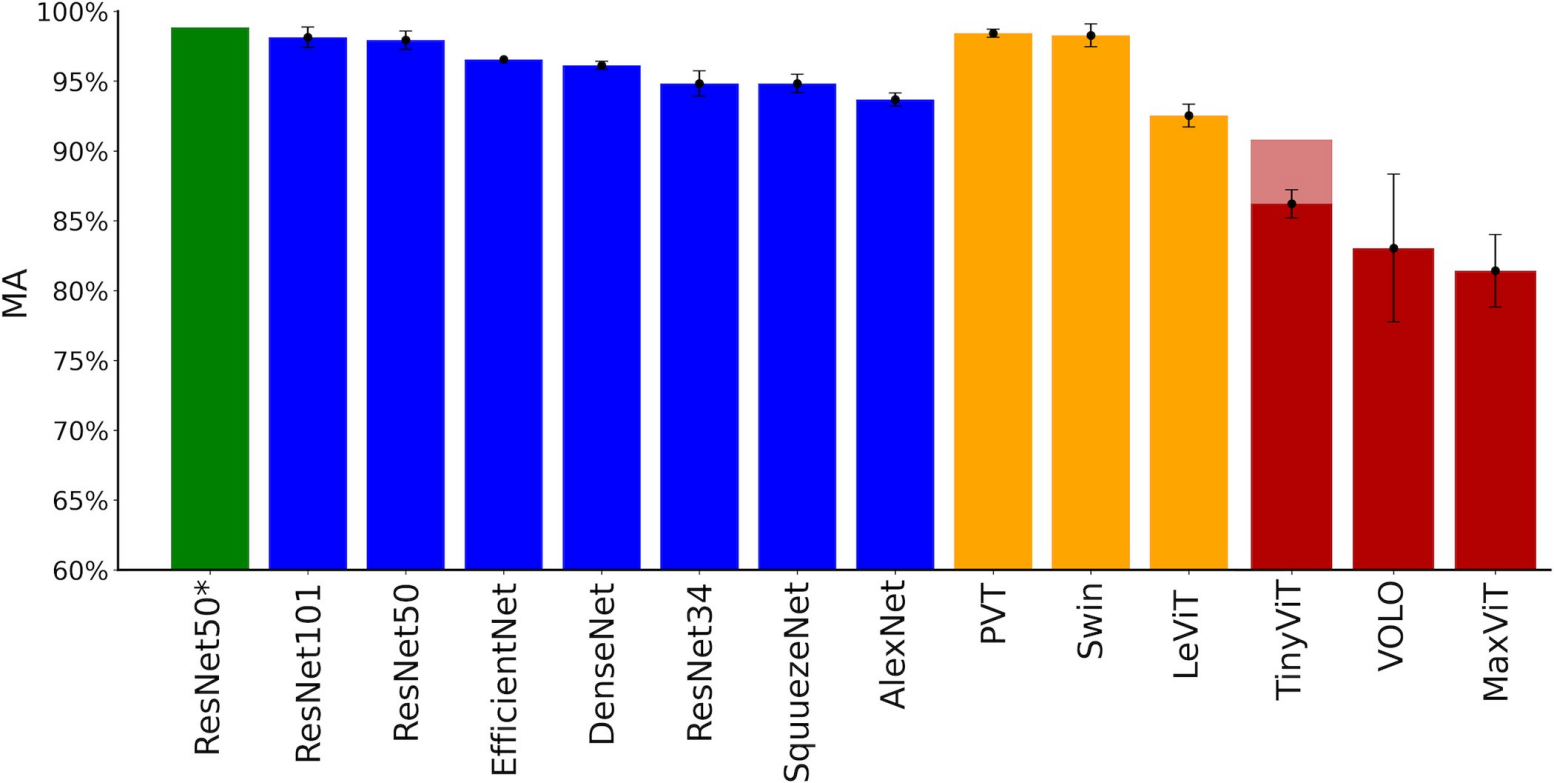
Mean *MA* for the various model architectures considered. The blue bars represent *CNN* model architectures, including *ResNet50*. The orange/red bars represent *ViT* model architectures. Within each model architecture, the error bars quantify the variability in *MA* performance between models generated by choosing different random seeds during training. The green bar represents the highest performing model within the *ResNet50* model architecture and ultimately becomes our chosen model. All models are trained using our multi-scaled tiling method, except for the red group models which are trained on the full images. While the red bars are trained using a batch size of 4 due to computational limitations, the faded red bar is trained with a batch size of 32.

In our model selection process, we investigate numerous deep learning model architectures (shown in Fig 3 and detailed in S3 Table). For each model test, we adjust the *PMF* Threshold to maximize the *MA*. Fig 3 illustrates the mean *MA* for various *CNN* model architectures, all of which use our multi-scaled tiling ingestion method (blue). The highest performing model within the *ResNet50* model architecture is plotted separately (green) because ultimately this becomes our chosen model. The figure also includes 3 *ViT* model architectures that use our tiling method (orange) and 3 *ViT* model architectures that are trained only on the full-scale image (red). We train *ViTs* in these 2 different ways to better compare and understand the capabilities of our chosen model. We find that the *ViTs* trained without our tiling approach consistently underperform compared to the *CNNs*. The purpose of training *ViTs* using our tiling method is to more directly compare their performances to the *CNN* architectures. Since *ViTs* already ‘tokenize’ an image into smaller chunks, their native data ingestion mechanism might be expected to reduce the impact of applying our tiling approach for these models. However, incorporating our tiling into *ViTs* nevertheless improves their *MA* performance. Taken together, these results highlight the suitability of using *ResNet50* coupled with our tiling ingestion method for classifying the all-over style of images in our data set.

For all the *CNN* model architectures trained in Fig 3 we chose a batch size of 64, 1 epoch, and an image size of 256 x 256 pixels and 3 color channels. The orange *ViT* architecture models use an image size of 224 x 224 x 3 (necessary to match the input architecture), but otherwise use the same hyperparameters as the *CNNs*. Additional model parameters are outlined in S2 Table. For training the red *ViTs*, we use a larger image size (512 x 512 x 3), 1 epoch, and a reduced ‘batch size’ of 4 because of computational limitations. We note that, because *tiny_vit* is a more computationally efficient *ViT* architecture, we are able to run models with a batch size of 32 and 2 epochs (after which there is no improvement in *MA*). This still results in an inferior performance (faded red top) when compared to *ResNet50*.

Ultimately, we choose *ResNet50* as our main model architecture over the other *CNN* architectures due to its consistently high *MA* and its established track record for a variety of image classification tasks [7,9,11,74]. We note that *ResNet50* exhibits comparable performance to *ResNet101* while requiring significantly fewer computational resources. We observe a notable improvement in model performance when using *ResNet50* compared to *ResNet34* with only a marginal increase in training time. We choose *ResNet50* over the *ViTs* considered due to *ResNet50*’s performance in Fig 3 and because its longer history of operation across society might be more compelling for the current art world.

We note that *ResNet50*’s performance deteriorates if we exclude our tiling process and simply train on the whole image: *MA* drops by 16.7% and 9.4% for batch sizes 4 and 64, respectively. To reach this higher performance, we explore different ‘voting’ schemes in terms of how the Pollock Signatures of each of the individual tiles would be used when calculating the *PMF*. Two example schemes include assigning each tile size a single ‘vote’ and giving each tile (irrespective of size) a single vote. The former approach yields a higher *MA*. Due to the prevalence of smaller tile sizes, the latter approach would be vulnerable to a dominance of small-scale characteristics. We also investigate giving different tile sizes a weighted ‘vote’ but we keep with the simpler equal voting scheme because the weighted approach doesn’t improve performance.

Finally, in terms of selecting which images are utilized in the validation set, we employ multiple random combinations to identify an effective validation set. This ensures robustness across different sets of images. While training on various validation sets yields good performance overall, we ultimately select the validation set that delivers the best results for *ResNet50*. This comprehensive approach allows us to fine-tune our model selection and achieve optimal *MA* when distinguishing between the Pollock and non-Pollock groups.

### Model generalization

**Fig 4:**
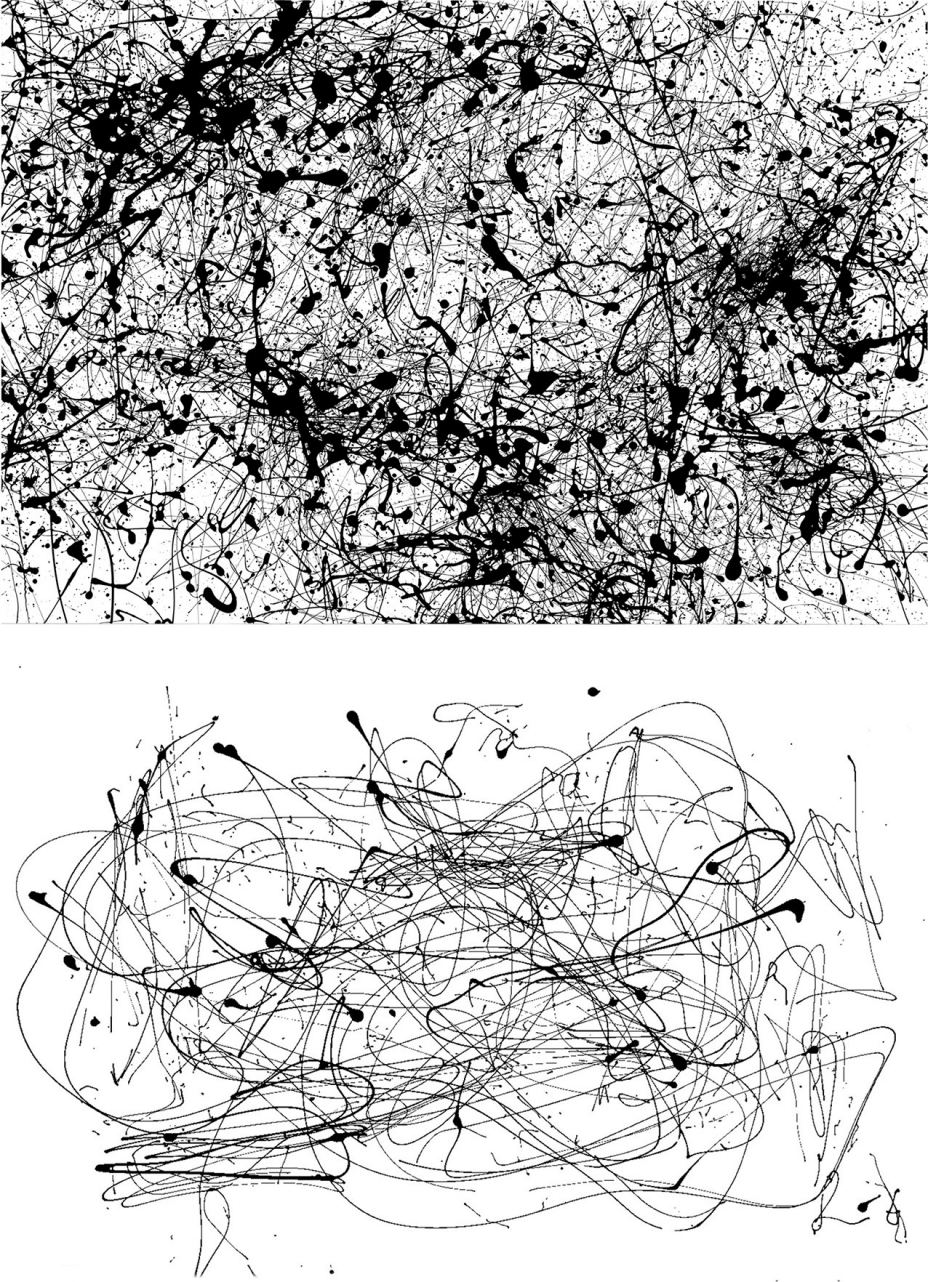
An example of an adult (top) and child (bottom) Dripfest image (61.5 x 91.5 cm).

In order to determine if our technique can generalize to other artists, we perform the following analysis. Rather than attempting to identify artworks by Pollock, we select another artist category from our collection of images and instead determine if our model can identify this category. We choose the Dripfest images for this test. These are a set of paintings created by 18 children and 32 adults in controlled settings. An example of each is shown in Fig 4. These novice painters attempted to imitate Pollock by pouring paint. We apply the same approach as before, but instead train our model to identify Dripfest paintings. To do this, we randomly sample a variety of images from our image collection, including authentic works by Pollock. This ensures a diverse selection of non-Dripfest paintings while maintaining a balanced training set.

One big difference from classifying Pollock artworks focuses on the number of paintings. Whereas we had 189 works in the Pollock category, the Dripfest category features 50. We therefore sub-sampled our data set of non-Dripfest images to achieve a balance of ‘imitation’ and ‘authentic’ paintings. Importantly, whereas approximately 50% of the paintings in our main study are by Pollock, only 16% of the images in our generalization test are by Pollock. Table 3 shows the image partitioning in more detail. Despite this much smaller number of images used in training, our machine performs exceptionally well with this alternative data configuration.

**Table 3:** Data partitioning of Dripfest and non-Dripfest artworks according to the number of artworks.

Data Partitioning (artworks)			
	Train	Validation	Hold Out
Dripfest	38 (76.0%)	9 (18%)	3 (6%)
Other	38 (55.1%)	11 (15.9%)	20 (29.0%)

Utilizing this new data set, we achieve a *MA* of 100% by using a Threshold for the Matching Factor (i.e the equivalent of *PMF*) of 0.85. This high accuracy is consistent with previous fractal investigations which successfully differentiated Dripfest paintings from Pollocks [17,31]. As we will see, our machine uses fractal content predominantly in its decisions. This result demonstrates that our model can be applied to other artists employing the pouring style.

## Results

### Pollock matching factors and The Pollock Dial

**Fig 5:**
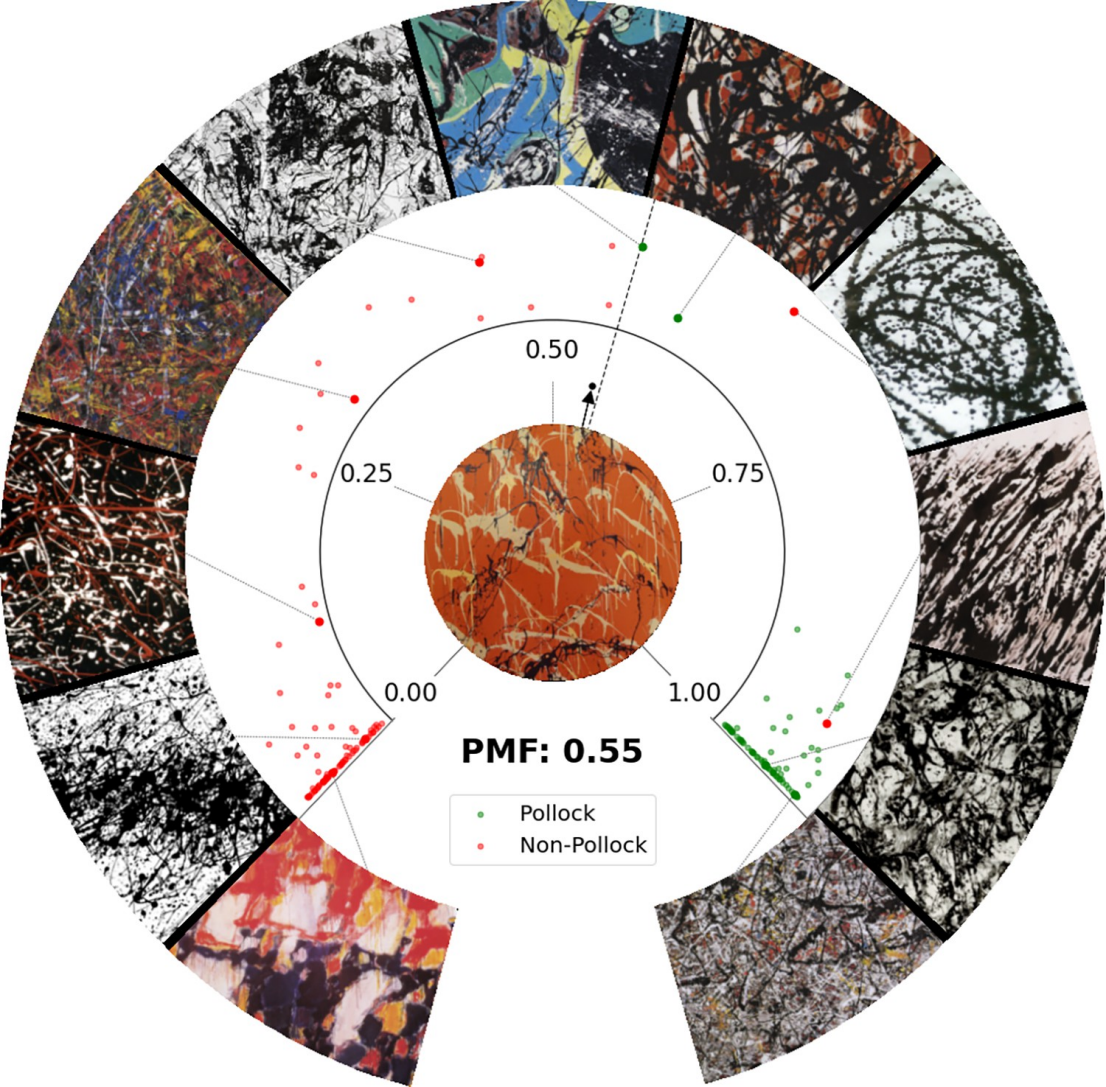
The Pollock Dial (see text for details).

The Pollock Dial is shown in Fig 5. The green dots represent Pollock poured works and the red dots represent artworks created by the other artists. Representative images of varying *PMF* values are shown in the outer circle for comparison. Moving clockwise around the Dial, the titles of these artworks are: *Untitled* (Sam Francis), *Adult 15* (Dripfest), *Untitled* (Wind Machine), *Abstraction Orange* (Jean-Paul Riopelle), *Picasso’s Guernica in the Style of Jackson Pollock* (Michael Baldwin), *Water Birds* (Pollock), *Free Form* (Pollock), *One* (Pollockizer), *Untitled* (Henri Michaux), *Enchanted Forest* (Pollock), and *Untitled Mural* (Pollock). The *PMF* Threshold is shown by the dashed radial line. We find that setting the Threshold at 0.56 generates the highest machine accuracy (*MA* = 98.9%) for distinguishing between the Pollock and non-Pollock groups. The image of a demonstration painting, *Dummy* (1504 x 112.8cm, by Richard Taylor), is shown at the Dial’s center (a full image is shown later in Fig 13). *Dummy* is selected from the non-Pollock validation set and serves as a useful demonstration because its mid-range *PMF* (0.55, represented by the black arrow in the inner circle) lies close to the Threshold.

The Pollock artworks with the lowest *PMF*s on the Dial are 2 of his earliest poured paintings—*Water Birds* from 1943 (0.56) and *Free Form* from 1945 (0.60). The majority of works by Pollock have the highest *PMF* value of 1. The non-Pollock artworks on the Dial have *PMF*s that span the range from 0 to 0.95. The Dial features only 2 images that the machine miss-classifies as Pollocks (i.e. that meet or exceed the Threshold). *One* was generated by ‘The Pollockizer’—a chaotic pendulum developed to generate Pollock imitations [35] (see Discussion). The other was *Untitled* by Henri Michaux (see Discussion). Whereas the Dial focuses on images in the inference set, we might also compare *PMF*s of paintings that are used in the training set when helpful. S4 Table provides a list of *PMF* values for all of the Pollock paintings.

**Fig 6:**
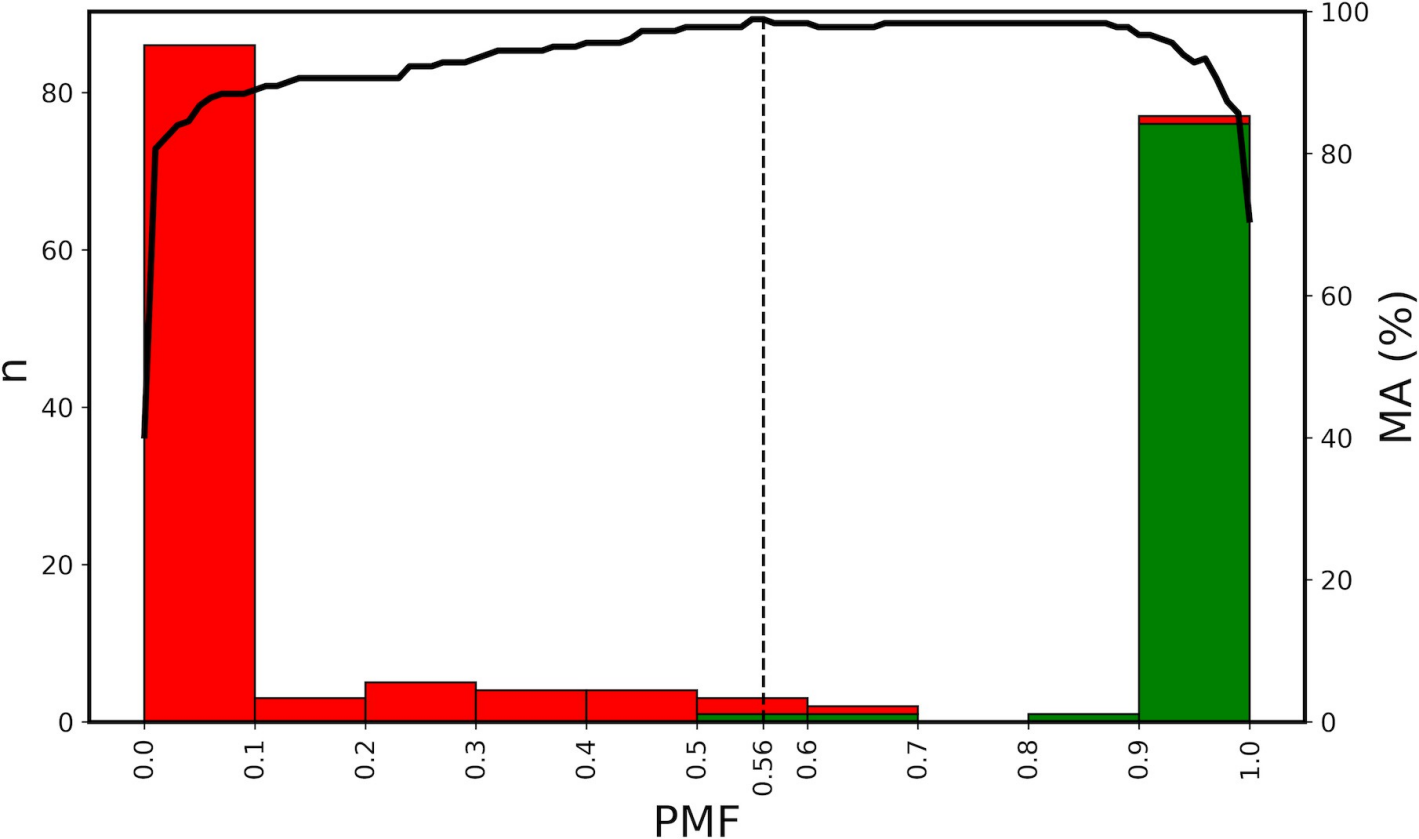
A histogram of the number of Pollock (green) and non-Pollock (red) images featured in the Pollock Dial. The number of images, *n*, in a given *PMF* range is plotted on the left y axis and the *PMF* values are plotted on the x axis. The black vertical line represents the chosen *PMF* Threshold. The black line is the *MA* obtained by shifting the threshold to different *PMF* values. These *MA* values are plotted on the right-hand y axis.

Because the 2 groups of artwork congregate mainly at the 2 extremes of the Dial (non-Pollocks at low *PMF* and Pollocks at high PMFs), many of the data points are superimposed. Because of this, in Fig 6 we show a histogram of the numbers of artworks, *n*, at various *PMF* values for the 2 groups. In this figure, we also show how the machine accuracy decreases as the *PMF* Threshold is moved away from its optimal value of 0.56. Because of the relatively few paintings with mid-*PMF* values, the accuracy remains fairly stable for mid-values and then deteriorates significantly at the very low and high values. In future research, we plan to calculate a machine confidence to express the probability of artworks having Pollock’s signatures based on *PMF* value. This will address the discrete character of the Threshold. However, it is clear from the histogram’s 2 distributions that the machine gradually gains confidence in a visual match to Pollock’s work as the *PMF* increases beyond the Threshold and then gains very high confidence close to *PMF* = 1. Similarly, the machine gradually gains confidence in a visual miss-match as the *PMF* decreases below the Threshold and then gains very high confidence close to *PMF* = 0.

#### Image quality confirmation

To increase robustness to variations in image quality and color variation, we use 2 sets of Pollock images: high quality color images and lower quality grayscale images (see Methods for more details). All images tested from both sets are classified correctly, with a mean *PMF* difference between the 2 sets of only 0.01. This small difference confirms that the technique is robust to reasonable variations in image quality. We also convert the color images to grayscale and find that the average *PMF* drops by only 0.02. This small difference indicates that the Pollock Signatures used by the machine are predominantly influenced by the patterns rather than the colors of the Pollocks’ features.

**Fig 7:**
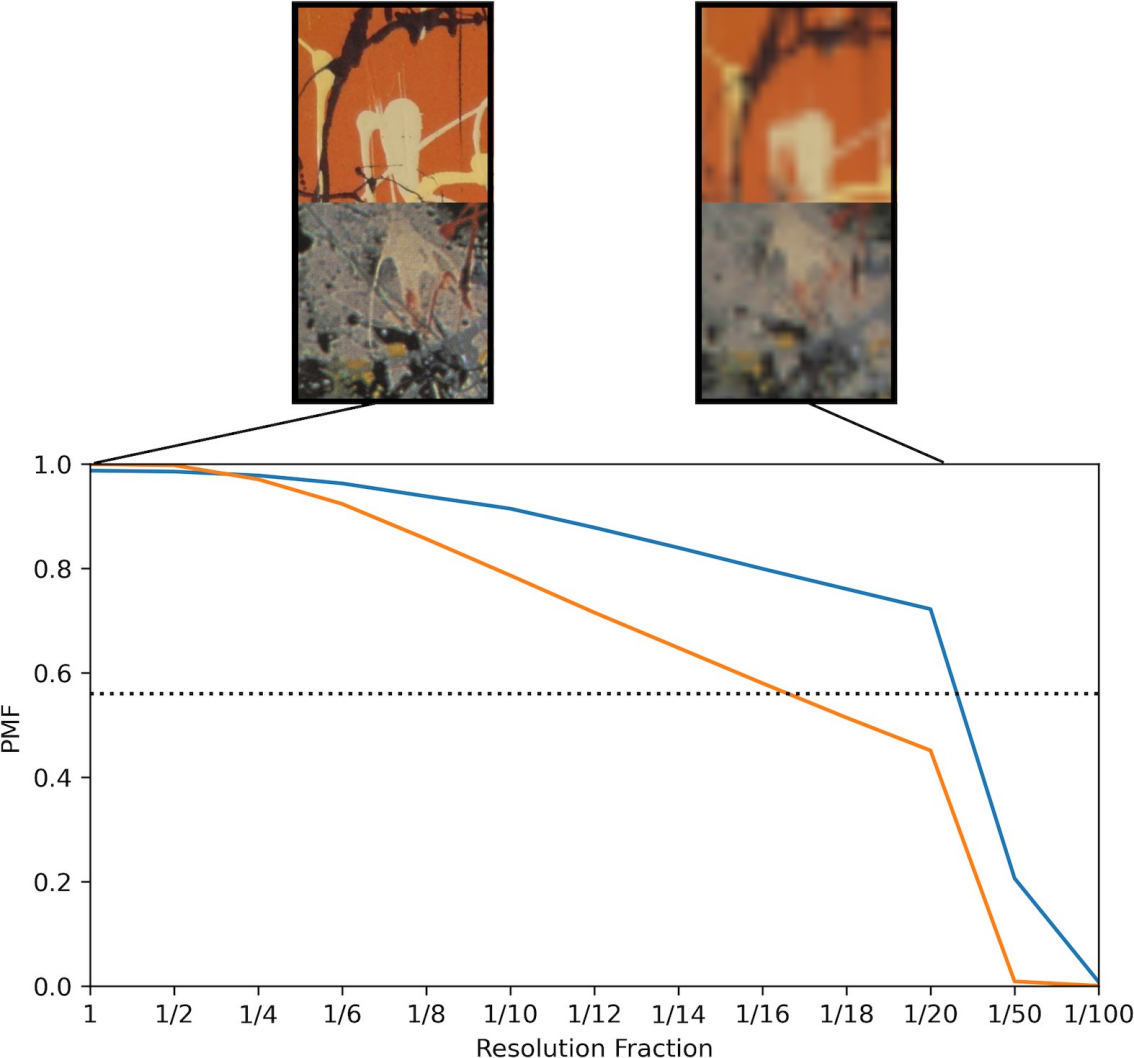
A Plot of *PMF* versus Resolution Fraction (*RF*) for the 2 sets of Pollock images: blue (high resolution, color images) and orange (lower resolution, grayscale images). The top images show close-ups of Pollock’s *Blue Poles* and *Dummy* at *RF* = 1 (scanned resolution, left) and *RF* = 1/30 (cliff resolution, right).

**Fig 8:**
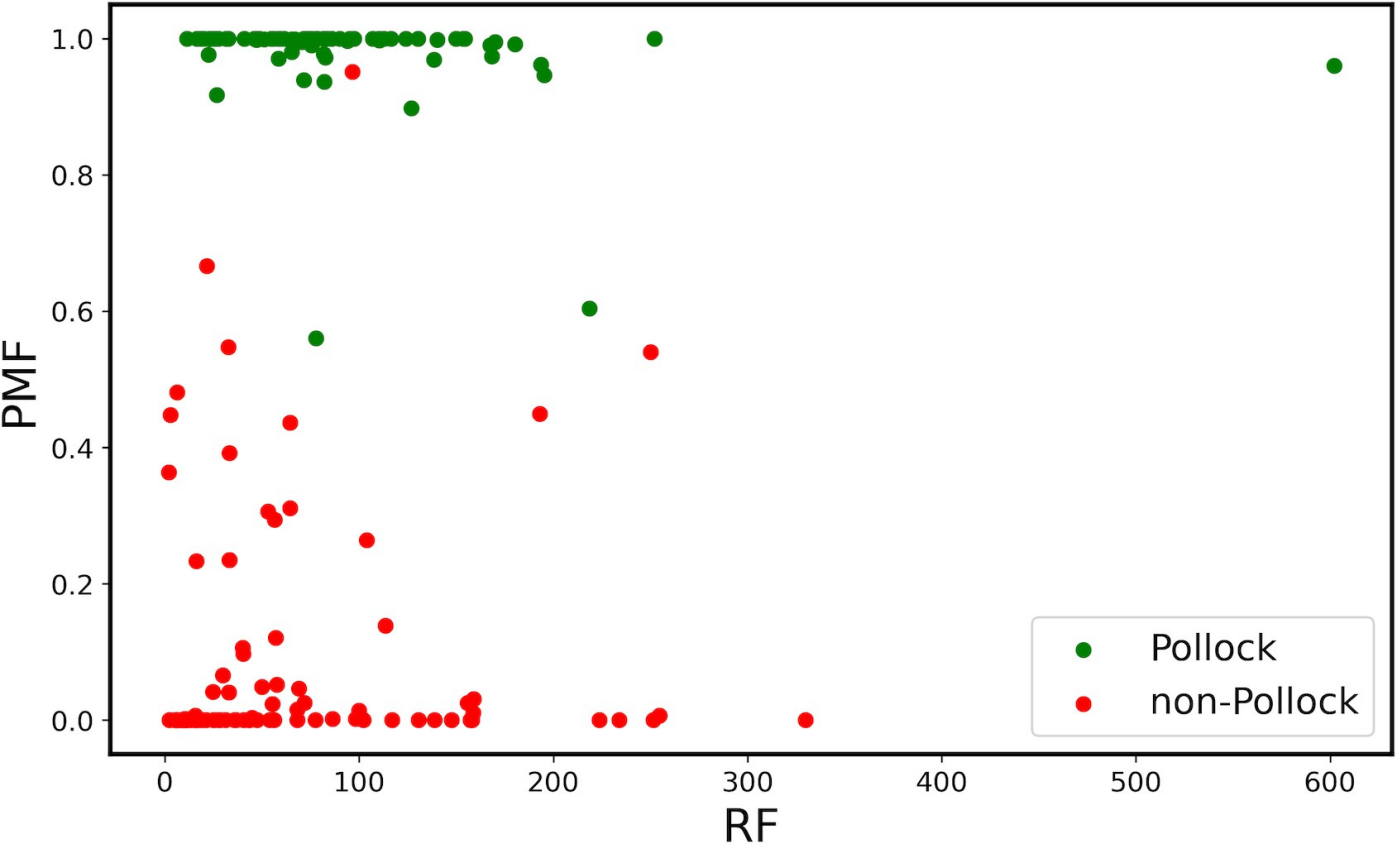
A plot of *PMF* versus *RF* for the Pollock (green) and non-Pollock (red) images on the Pollock Dial.

In Fig 7, we show the deterioration in *PMF* that occurs as we artificially reduce the image resolution for the high resolution, color images and lower resolution, grayscale images. We plot the Resolution Fraction (*RF*) on the horizontal axis. *RF* = 1 corresponds to the scanned resolution whereas, for example, *RF* = 1/30 corresponds to decreasing the pixel resolution along each direction (width and height) by a factor of 30. When the colored lines drop below the horizontal line (indicating the *PMF* Threshold of 0.56), we refer to this as falling off the ‘resolution cliff.’ At this point, the resolution decrease distorts the Pollock Signatures so much that the machine will incorrectly classify Pollocks as imitations. The orange line falls off the resolution cliff at a lower *RF* than the blue line. For both lines, the scanned resolution (*RF* = 1) lies well away from the cliff. The top images show close-ups of Pollock’s *Blue Poles* and *Dummy* to demonstrate the drastic resolution reduction needed to fall off the cliff. More generally, *PMF* is not correlated with pixel resolution (*PR* is the pixel density using physical measurement units, i.e. of the artwork rather than its image) when we look across all Pollock and non-Pollock images (Fig 8).

**Fig 9:**
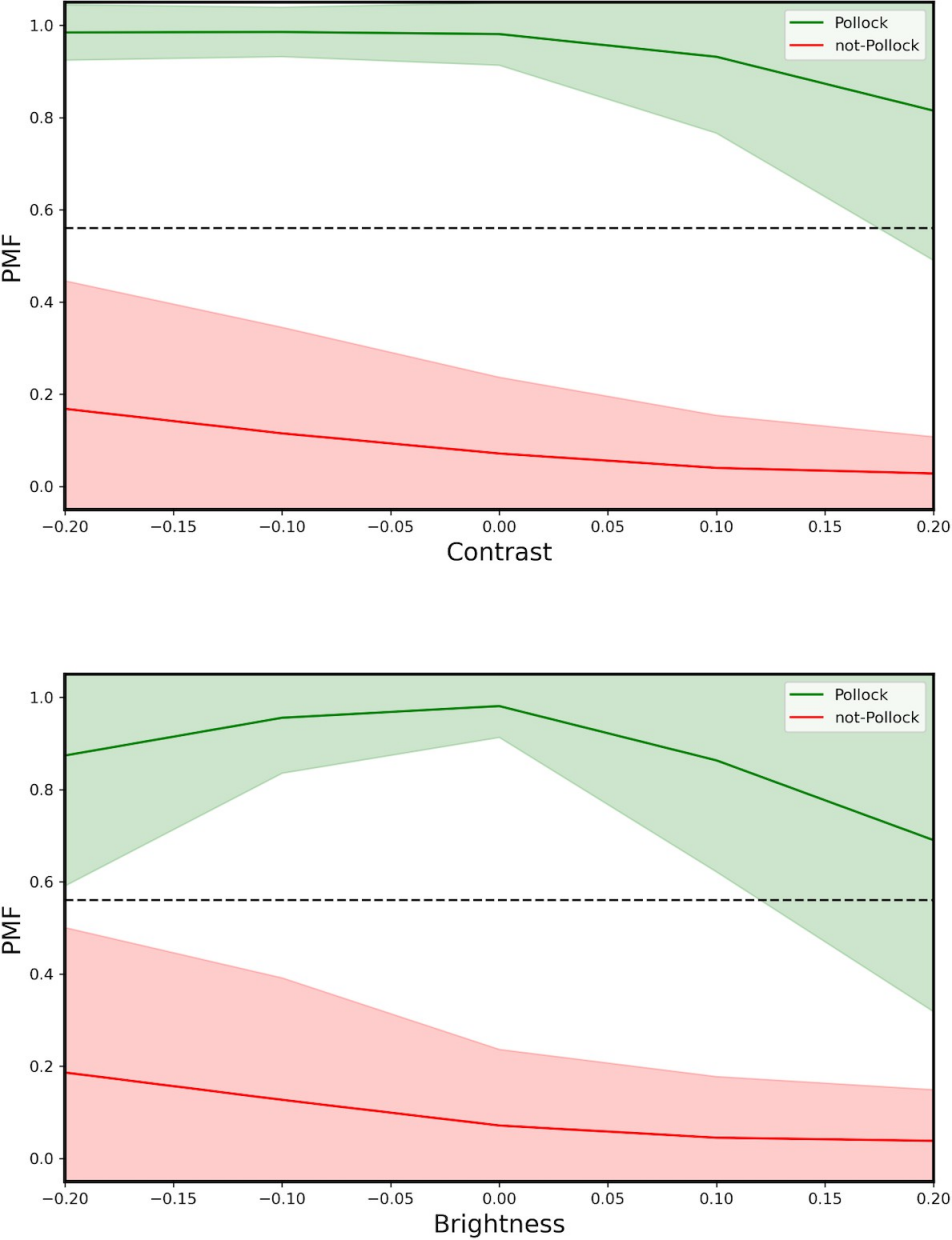
Average *PMF* plotted against the image contrast (top) and image brightness (bottom) for the Pollock (green) and non-Pollock images (red). The broad colored regions correspond to the standard deviations in the data. The horizontal line represents the *PMF* Threshold. In each case, 0 corresponds to the undistorted value on the x axis.

In Fig 9, we plot the mean *PMF* for the Pollock and non-Pollock images and show how these values deteriorate when we artificially change the image contrast (top) and image brightness (bottom). In each case, we restrict the changes to the range of values relevant to human viewing. Pollock works feature a web of multiple interacting layers of paint that tend to generate low contrast, low brightness images. It is therefore expected that, for example, artificial boosts to contrast and brightness will be accompanied by a drop in *PMF* and that, equivalently, these might increase the *PMF* of imitations. Nevertheless, Fig 9 demonstrates that these changes do not cause the *PMF* values of the non-Pollocks to cross the Threshold (and therefore do not impact *MA*) provided images stay within the reasonable (i.e. natural) levels of brightness and contrast.

#### The Zoom-in Charts

**Fig 10:**
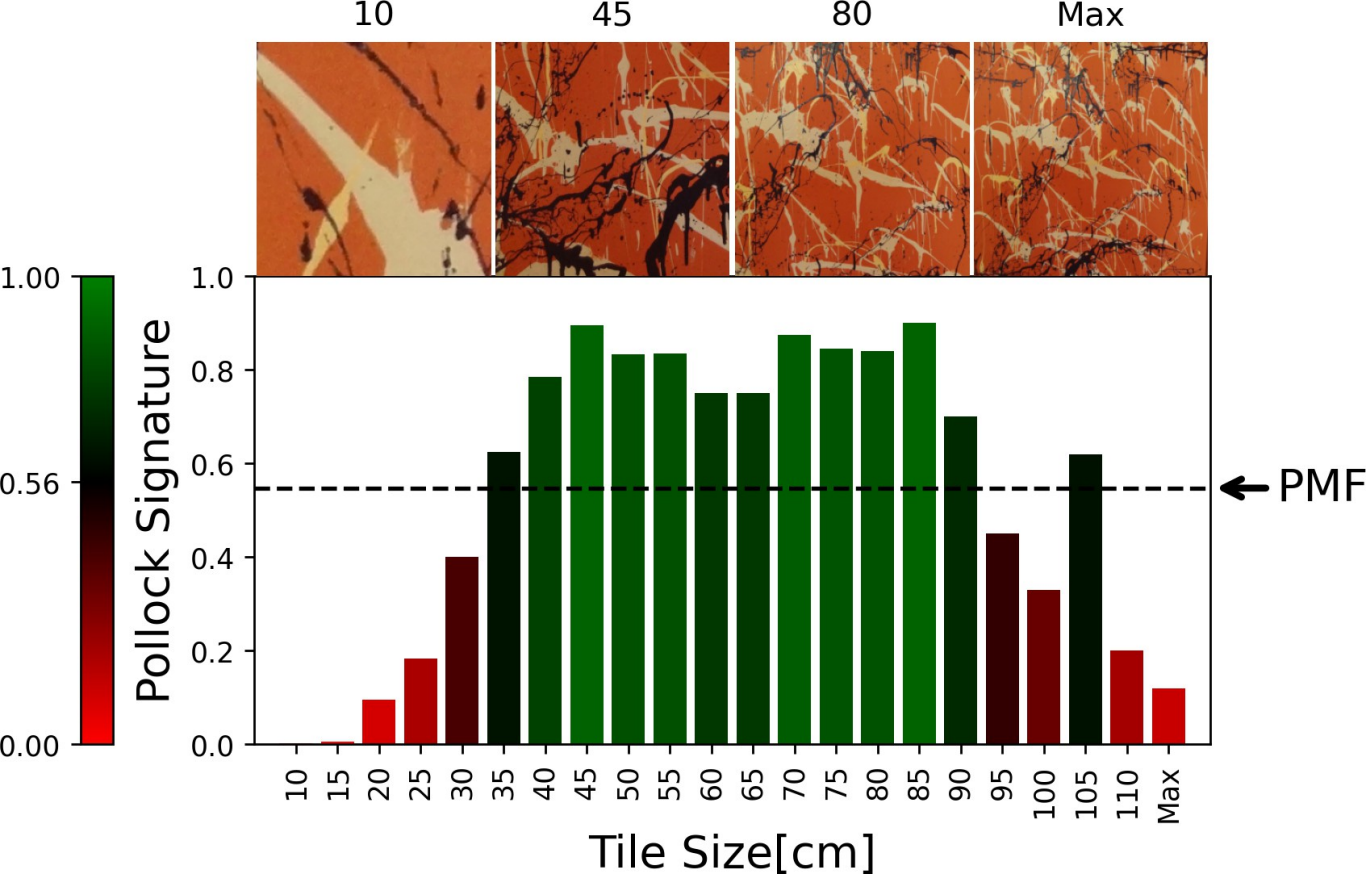
Zoom-in Chart for *Dummy* (see text for details). The images above the Chart show example tile images.

In Fig 10, we show the Zoom-In Chart for *Dummy*. Its *PMF* value is indicated in the Chart by the right hand arrow pointing at the dashed line. The vertical bar to the left of the Chart represents the color variations shown in the Chart. Tile sizes with a Pollock Signature below the *PMF* Threshold are shaded red while those above are shaded green, with the shade of the color darkening as the Threshold is approached. The Chart therefore allows us to identify size scales that deviate away from Pollock’s style based on these color variations. For *Dummy*, *SI* = 0.37 and *M* = 11.00.

**Fig 11:**
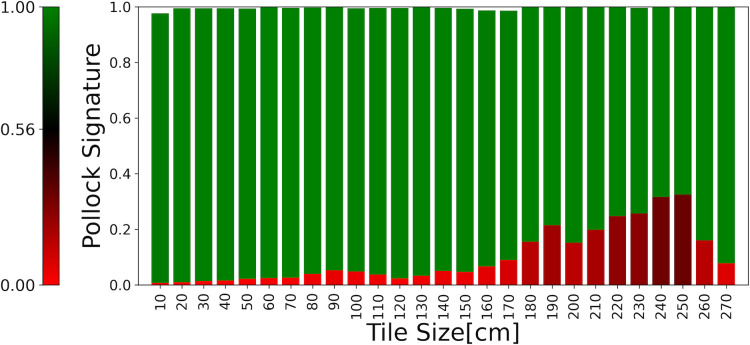
The Zoom-In Chart averaged across all non-Pollock poured paintings superimposed on the Zoom-In Chart averaged across all Pollock poured paintings.

In Fig 11, we superimpose the Zoom-In Chart averaged across all non-Pollock poured paintings on the Zoom-In Chart averaged across all Pollock poured paintings. The Charts consider tile sizes up to the maximum tile size found in a Pollock painting (270 cm). Whereas the Pollock tile averages are all green, the non-Pollocks are all red. Furthermore, whereas the Pollocks reveal the scale invariance expected for fractals, this is reduced for the non-Pollocks.

**Fig 12:**
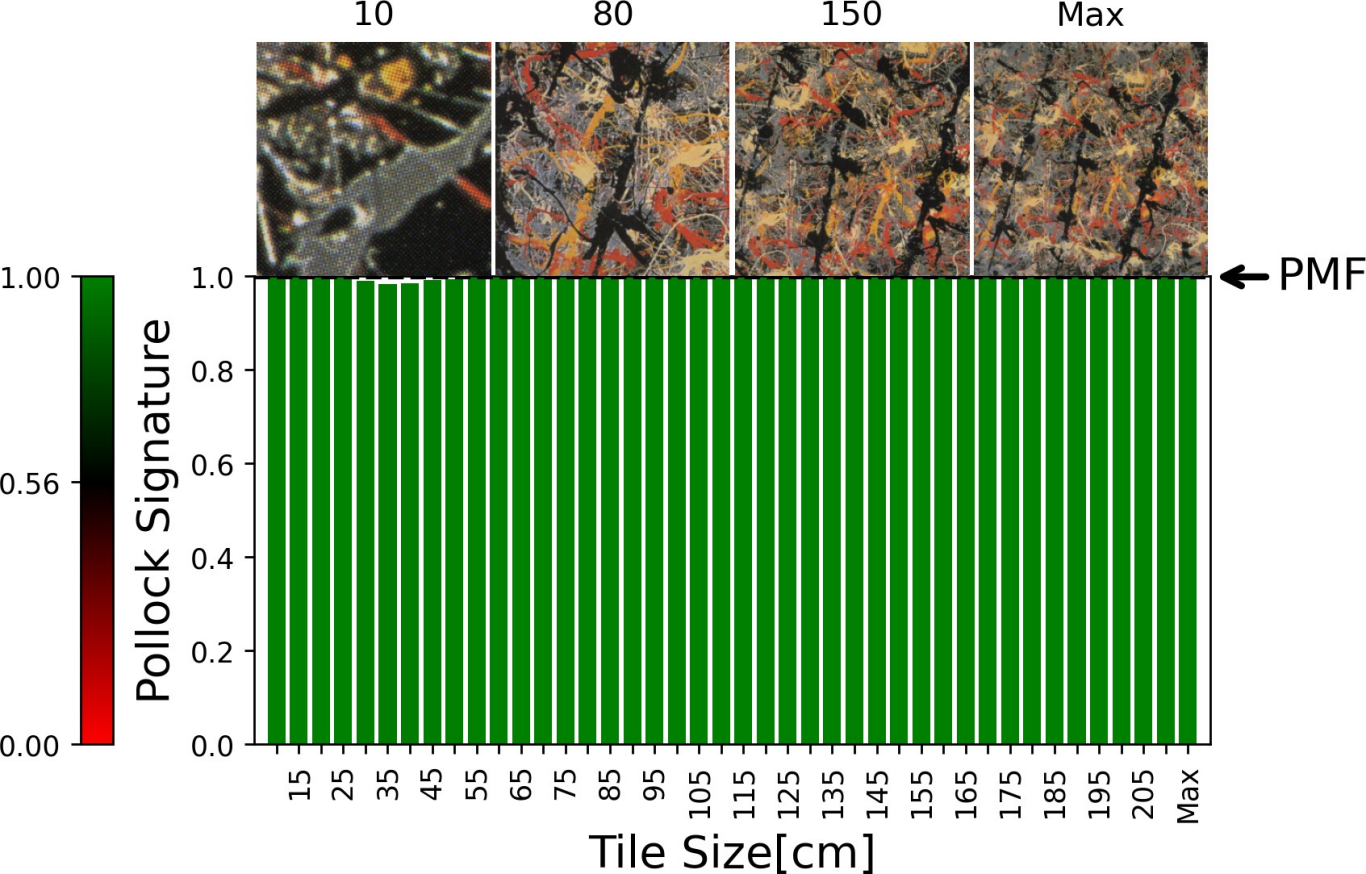
The Zoom-in Chart for *Blue Poles* (see text for details). The images above the Chart show example tile images.

To compare *Dummy*’s Zoom-in Chart to Pollock’s work, in Fig 12 we show the Zoom-In Chart for *Blue Poles*, which is quantified by *SI* = 0.99 and *M* = 21.00. As expected, the Zoom-in Chart reveals all tile sizes to be green for *Blue Poles*—the average Pollock Signatures are consistently close to 1 at all tile sizes.

#### The signature maps

**Fig 13:**
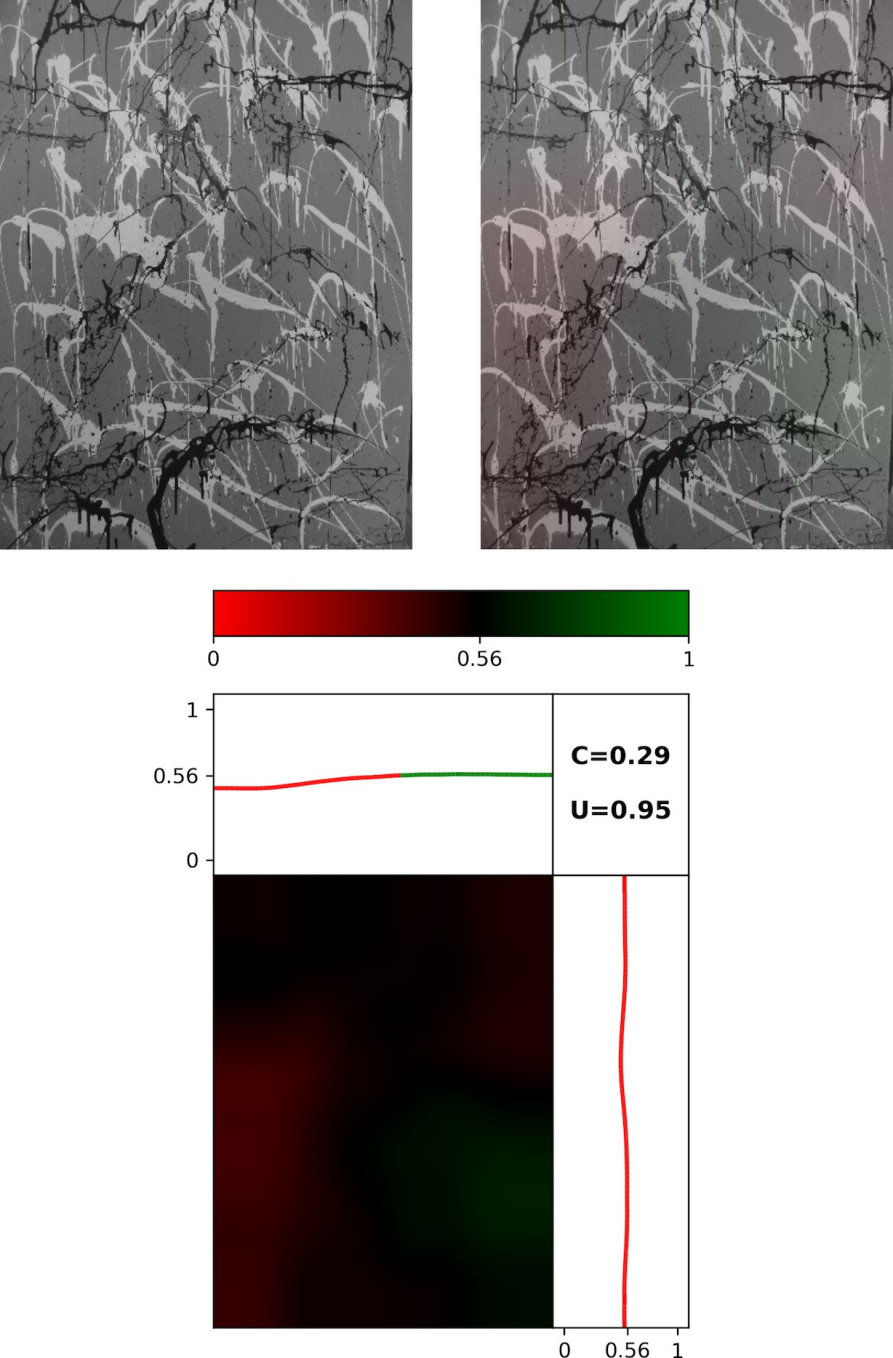
Spatial investigation of *Dummy’s* Signatures. Grayscale image of *Dummy* (top left panel) and the same image with the Signature Map overlaid (right panel). The bottom panel focuses on *Dummy*’s Map. The panel above the Map plots the average Pollock Signature as we move from left to right across the canvas and the panel to the right does the same as we move from bottom to top.

In Fig 13, we show a grayscale image of *Dummy* (left panel) and the same image with the Signature Map overlaid (right panel). The bottom panel focuses on *Dummy*’s Map. The color bar represents the color variations used in the Map. Locations with an average Pollock Signature below the *PMF* Threshold are shaded red while those above are shaded green, with the shade of the color darkening as the Threshold is approached. The Map therefore allows us to identify regions of the artwork that deviate away from Pollock’s style based on these color variations. The panel above the Map plots the average Pollock Signature as we move from left to right across the canvas and the panel to the right does the same as we move from bottom to top. The Map for *Dummy* is quantified by *U* = 0.95 and *C* = 0.29.

**Fig 14:**
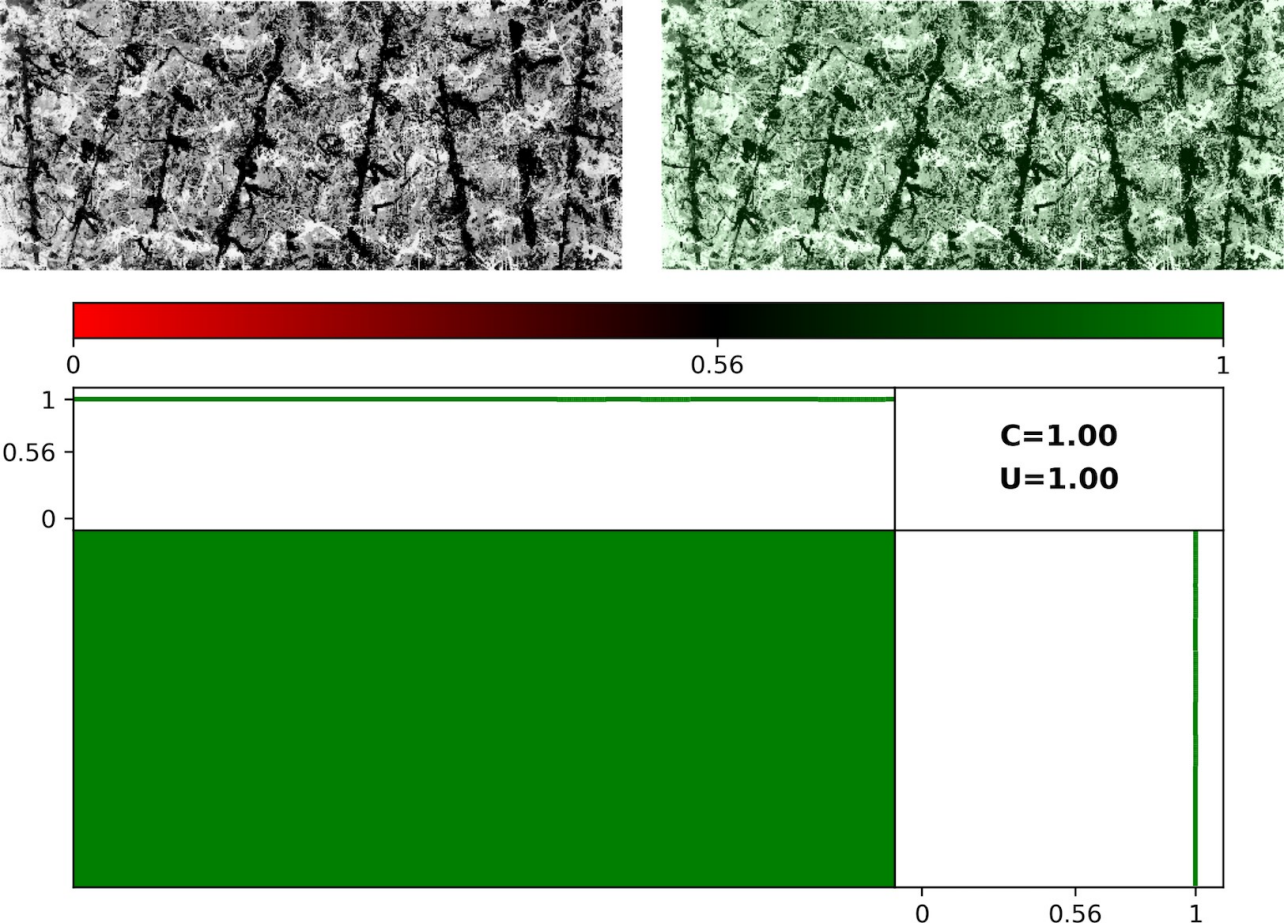
Spatial investigation of *Blue Poles’s* Signatures. Grayscale image of *Blue Poles* (top left panel) and the same image with the Signature Map overlaid (right panel). The bottom panel focuses on *Blue Poles*’s Map. The panel above the Map plots the average Pollock Signature as we move from left to right across the canvas and the panel to the right does the same as we move from bottom to top.

To compare *Dummy’s* Signature Map to Pollock’s work, in Fig 14 we show the equivalent images for *Blue Poles*, which is quantified by *U* = 1.00 and *C* = 1.00. As expected from his all-over style, the Signature Map is uniformly green and the Signatures are close to 1 for all widths and all heights. Significantly, the famous 8 ‘poles’ painted within the art work are sufficiently splattered that they have high Signatures and do not disrupt the uniformity of the Map.

**Fig 15:**
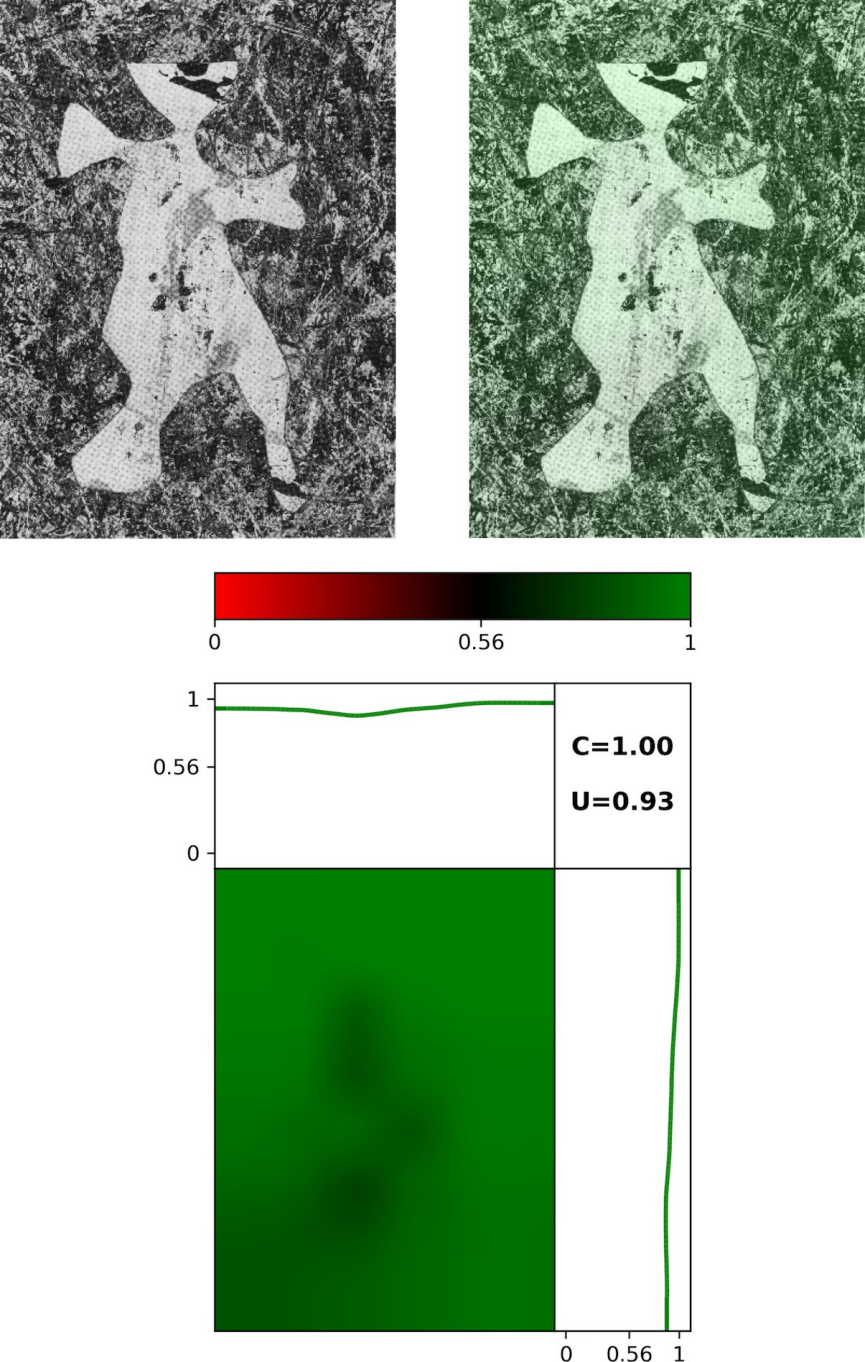
Spatial investigation of Pollock’s *Untitled*: *Cut-Out* (77.3 x 57cm, Ohara Museum of Art, Japan). Grayscale image of *Cut-out* (top left panel) and the same image with the Signature Map overlaid (right panel). The bottom panel focuses on *Cut-Out*’s Map. The panel above the Map plots the average Pollock Signature as we move from left to right across the canvas and the panel to the right does the same as we move from bottom to top.

In Fig 15, we show a Map for Pollock’s *Cut-Out* (1948) which has an unusual artistic feature—the shape of a human figure has been removed from the center of the painting. The machine detects this region successfully, as indicated by the darkened region at the center of the Map. The drop in Pollock Signature in this central region is also apparent in the left-right and bottom-top plots. Accordingly, *U* = 0.93 and *C* = 1.00 are lower than for *Blue Poles*. Significantly, the Pollock Signatures in the regions surrounding the cut-out are high and these ensure that the *PMF* = 0.92 lies above the *PMF* Threshold and that the machine correctly classifies the painting as having the visual style of a Pollock.

## Discussion

### The all-over style and pollock timeline

We declared 3 scientific goals in the Introduction. The first and second are linked: can we achieve a high *MA* for distinguishing Pollocked poured works by integrating a robust, established machine model with a novel image ingestion approach based on multi-scaled tiles? Our resulting high value, *MA* = 98.9%, demonstrates the power of machine learning for future Pollock authenticity studies when its results are combined with other techniques such as human vision inspection, provenance investigations, and materials analysis. The high *MA* also provides scientific evidence that Pollock’s artistic signature is quantifiably different to those of other artists who adopt his technique of pouring paint. Pollock’s contributions to modern art therefore go beyond making the pouring technique famous. He should be celebrated for his specific form of pouring paint. Our third scientific goal probes this specific form by asking the following question—can we develop novel visual aids and associated interpretive parameters to move beyond the ‘black box’ character of *MA* by relating the machine’s Signatures to the artistic development of Pollock’s all-over style?

**Fig 16:**
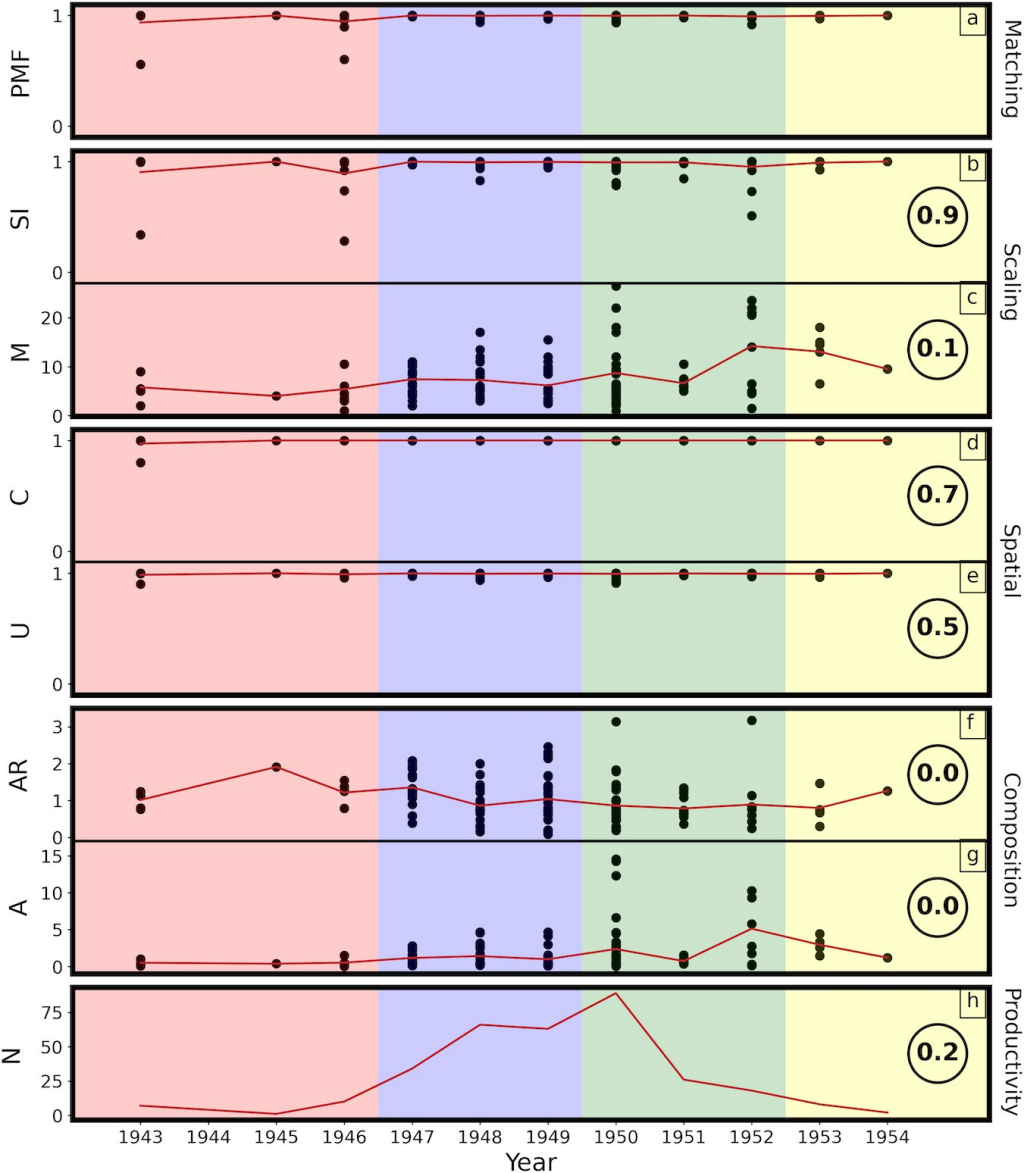
A timeline chart of the evolution of the various machine parameters (*PMF*, *SI*, *M*, *C*, *and U*) along with other artwork parameters (*N*, *A*, and *AR*) spanning from 1943 to 1954. See text for details.

In Fig 16, we chart the evolution of the 5 machine parameters (*PMF*, *SI*, *M*, *C*, *and U*), beginning with his first poured work in 1943 through to his final poured work in 1954. *SI* and *M* are grouped together as scaling parameters: *C* and *U* are grouped together as spatial parameters. In addition to the machine parameters, we also chart changes in Pollock’s productivity (*N* is the number of paintings per year) and composition (*A* is the canvas area and *AR* is its aspect ratio). In each case, the black dots represent individual paintings (many of which are superimposed) and the red lines represent the average values for each year.

The colored backgrounds represent the phases of his development from an art history perspective: ‘preliminary’ (pink), ‘transitional’ (purple), ‘classic’ (green), and ‘final’ (yellow) [1]. His ‘preliminary’ works are composed of a few poured trajectories superimposed on a dominant brush-worked background. His transitional works feature a growing contribution of interacting poured trajectories dominating over the brushwork. His ‘classic’ works are characterized mainly by poured trajectories. His ‘final’ works mark a fall off from his large, densely poured masterworks of 1950–52. Intriguingly, there is little variation in *PMF* with year. Aside from some very early variations, his distinguishing characteristics remain constant through the years. This implies that, although the variations between the 4 phases are significant for charting Pollock’s visual development, they all display Pollock’s ‘hand’. The differences in Pollock’s evolution are subtle when compared to the differences between Pollocks and non-Pollocks.

The circled value to the right of each panel in Fig 16 quantifies the correlation between the *PMF* and each of the parameters, where 0 corresponds to no correlation (i.e. the relationship is random) and 1 corresponds to the strongest correlation. Interestingly, there is little correlation between *PMF* and most parameters (*N*, *A*, *AR*, *M*). This indicates that Pollock’s Signature remains strong as these characteristics vary across the paintings. Notably, Pollock’s Signature has no correlation with the canvas shape and size that he chose to work on. Taken together, this implies that the presence of the canvas edge has little impact on his Signature. This is consistent with traditional descriptions of his painting process. These picture Pollock as painting beyond the confines of his canvas, with the studio floor capturing the paint trajectories that extend beyond the artwork. There is also only a very weak correlation with the number of paintings he created at a given time. This is intriguing because at this peak in 1950 Pollock created some paintings in parallel rather than consecutively. There has been speculation concerning the impact of this switching between works on their appearance. Our result suggests that his Signature is strong whether it arose from his peak production or from rarer works painted in isolation.

**Fig 17:**
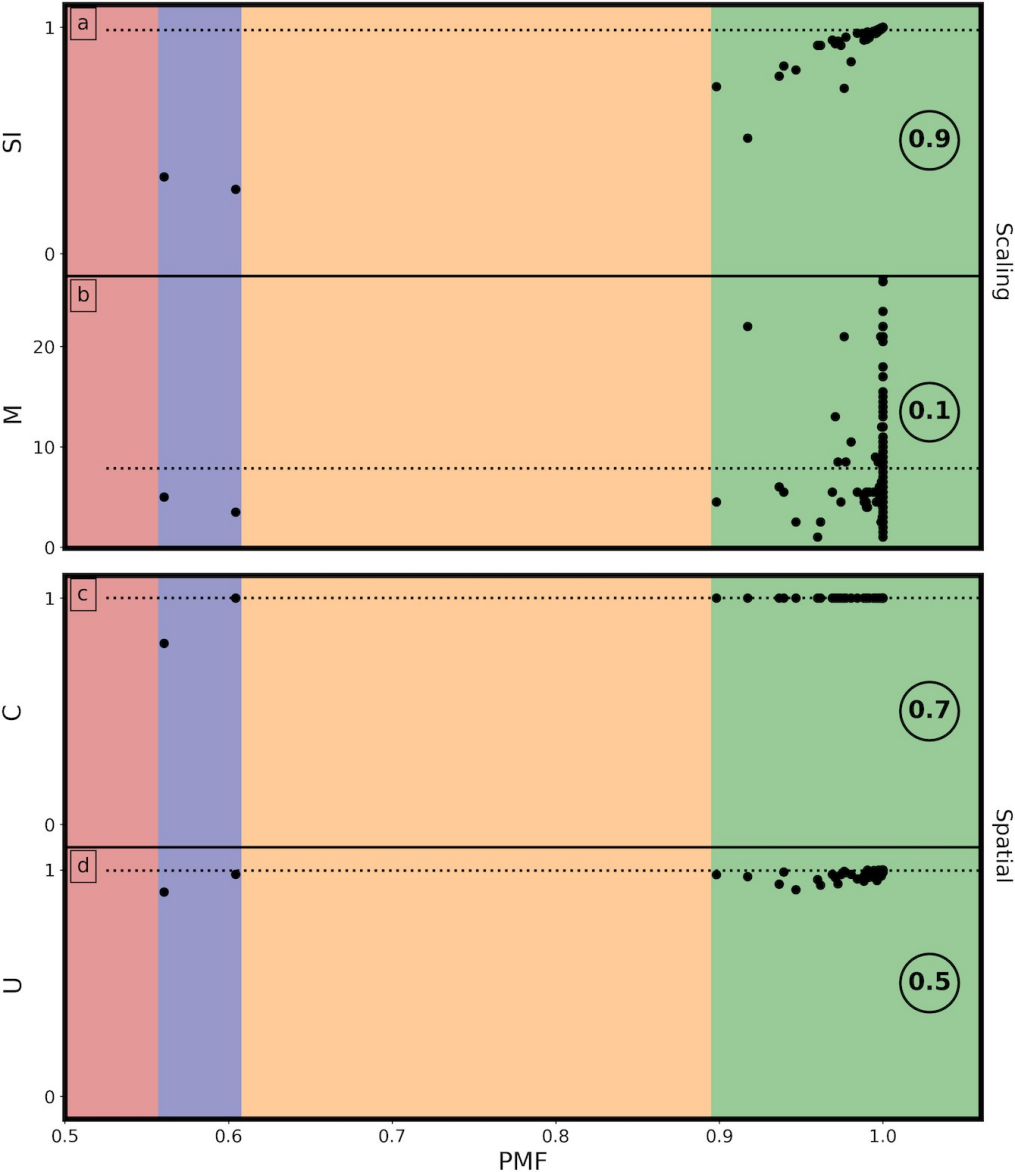
Plots of *SI*, *M*, *C*, *and U* against *PMF* (see text for details).

To examine the correlation with the 4 machine parameters in detail, Fig 17 plots their relationship with *PMF* directly. The colored backgrounds are used to highlight the *PMF* ranges that are most populated by the artworks, and the dashed horizontal lines denote the mean values of *SI*, *M*, *U*, and *C*. A medium strength correlation can be seen between *PMF* and the spatial characteristics (*U*, *C*) of the artworks, consistent with Pollock’s all-over style. The low correlation between *PMF* and *M* can be expected from the low correlation with canvas size—small canvases offer little opportunity for zooming out from the small to large tiles. Strikingly, there is a very strong correlation between *PMF* and *SI*, consistent with the fractal character of Pollock’s work. In addition to examining the correlations of the bulk of Pollock’s work, Fig 17 also provides clarity for why the images of Pollock’s *Water Birds* (*PMF* = 0.56) and *Free Form* (*PMF* = 0.6) appear at the center of the Pollock Dial. Although their *U*, *C*, and *M* values appear close to the mean values, their *SI* values lie well below. This suggests that the poor fractal scaling of these images distinguishes them from the rest of Pollock’s work. Below, we will examine the key elements of fractality and uniformity of Pollock’s work in more detail.

### Pollock’s all-over style

Although Pollock is famous for the spatial uniformity of his all-over style, this quality of his work hasn’t previously undergone a detailed examination using quantitative image analysis techniques. The above results support the traditional picture of his all-over style. The mean values of *C* and *U* are both close to 1, indicating that expansive regions of the canvas surface have very strong Signatures (colored green in the Maps) and, accordingly, there is little variation in these Signatures across different locations. Our Tests involving changes in canvas orientation are further consistent with his all-over style. The average *PMF* values at different orientations (rotations along with vertical and horizontal flips of the canvas) of the Pollocks in the inference set do not vary from the average *PMF* measured in the original orientation.

The Pollock Maps shown in Figs 14 and 15 emphasize their value as interpretational tools. *Blue Poles* serves as a powerful demonstration of the spatially-uniform Signature of his all-over style. In particular, the 8 poles are sufficiently splattered that they do not impact the uniformity of the Signatures nor the overall *PMF*. This effect is seen in other examples of his work. For example, *Comet* features a long white line stretching from top to bottom, and *The Deep* features a large dark region at its center. These features do not disturb the associated Maps because of their splattered character. More extreme deviations, such as the cut-out shape in Fig 15, result in a darkening of the map in the impacted region. However, the high Signatures in the surrounding regions ensure a high *PMF*. This is seen in other examples of his work. For example, *The Wooden Horse* features a wooden object that darkens the impacted region but doesn’t dip the *PMF* below the Threshold.

**Fig 18:**
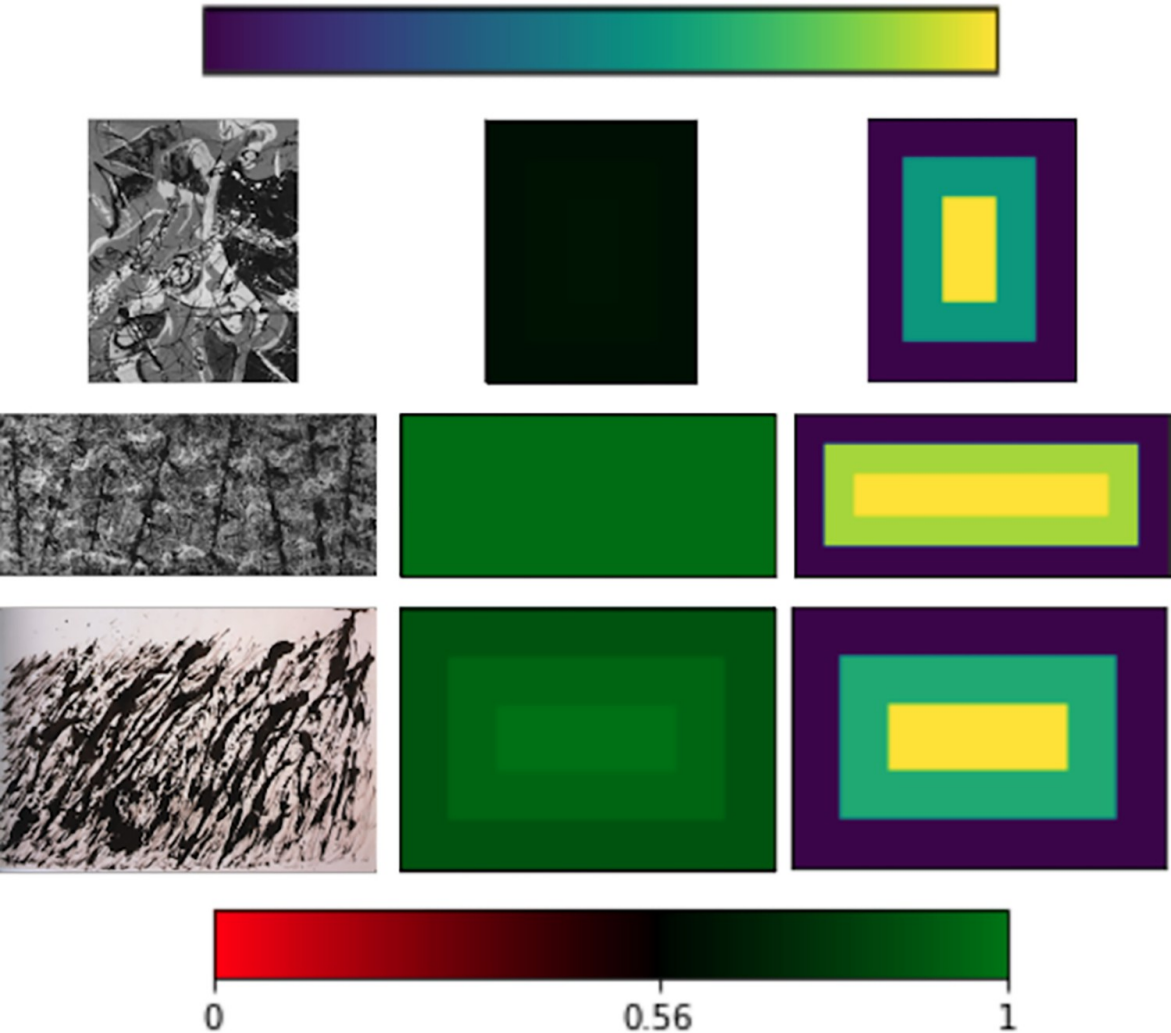
Radial maps for Pollock’s *Water Birds* (66.4 x 53.8cm, Baltimore Museum of Art, USA) (top row), Pollock’s *Blue Poles* (middle row), and Henri Michaux’s *Untitled* (74.9 x 107.9cm, Edward Thorp Gallery, New York) (bottom row). The left column shows the artwork, the middle column shows the radial map plotted using the absolute colors, and the right column shows the radial map plotted using relative colors. The top and bottom bars show the relative and absolute color ranges, respectively. See text for details.

Returning to the role of the canvas edge, Fig 18 shows radial maps for 3 example paintings—*Water Birds* (one of Pollock’s first poured works, appearing close to the Threshold with *PMF* = 0.56), *Blue Poles* (an exemplar of Pollock’s all-over style with *PMF* = 1) and Henri Michaux’s *Untitled* (a rare example of a non-Pollock work lying above the Threshold with *PMF* = 0.95). The middle and right columns show radial maps that have been divided into 3 regions (inner, middle, and outer) and the mean Signature averaged across each region is plotted. The middle column is plotted using the absolute colors (i.e. as used in the standard Maps and shown in the bottom bar). The right column shows relative colors to highlight small changes. For *Water Birds*, the Signature varies from 0.607 (purple) to 0.632 (yellow), *Blue Poles* varies from 0.997 to 0.998, and *Untitled* varies from 0.897 to 0.986. These values emphasize the subtle relationship between *PMF* and ‘all-overness’: *Untitled* has a higher *PMF* than *Water Birds*, even though it has a larger radial variation in its Signature.

Intriguingly, all 3 paintings display a systematic deterioration in Signature as the canvas edge is approached. This indicates that even if Pollock continued to paint beyond the artwork’s intended boundary, the boundary nevertheless had a subtle effect on his pouring technique. There are clues that Pollock sometimes spotted this deterioration and took measures to hide it—in particular, for some works he folded parts of the painted canvas behind the work to highlight the central region. Clearly, this wasn’t necessary for *Blue Poles*, where our results show the deterioration is minimal. This deterioration effect is consistent with our decision to use the poured patterns on Pollock’s studio floor (i.e. the heavily deteriorated pattern formed by the trajectories that completely missed the canvas) as a non-Pollock pattern in the training process (see later).

### Pollock’s fractal scaling

In addition to the strong correlation between *PMF* and *SI* shown in Figs 16 and 17, the comparison of the Zoom-in Charts in Fig 11 further emphasizes the scale invariance of Pollock’s work. Pollock’s Signature is consistently scale invariant and the rare works that show variations in scale can be identified by their fall off in *PMF* value. We note that the previously published fractal model of Pollock’s work pictures 2 distinct pattern generation processes. Whereas the balancing motions of his body are proposed to generate one set of fractals dominating at size scales above the transitional size of approximately 5cm, the spattering of the fluid paint is proposed to generate a second set of fractals at smaller scales [17]. Because our machine process focuses on tile sizes starting at 10cm, it should be sensitive to Pollock Signatures generated by the balancing motions. However, we note that for several Pollocks (these include *Number 22* (1950), *Number 24* (1950), *Brown and Silver II* (1951), and *Untitled* (1952)) we do see that the Pollock Signatures at the 10cm tile size are smaller than those for the larger tile sizes, suggesting that perhaps the spattering process might have started to dominate at the 10cm tile size for these paintings.

In addition to considering the degree of scale invariance of the patterns, fractal studies also look at scaling parameters (such as fractal dimension *D*) that quantify the rate at which the patterns shrink with magnification [12]. The fractal model of Pollock’s work introduced distinct fractal dimensions to quantify the scaling behavior of the 2 size regimes—*DD* for the ‘drip’ process occurring below 10cm and *DL* for the ‘Levy’ body motion process occurring above 10cm. The research found that *DL* on its own was insufficient for distinguishing Pollocks from non-Pollocks [17]. Because our machine focuses on sizes of 10cm and larger, we would therefore expect to see little correlation between *PMF* and *DL*. When we employ *DBC* and *SDBC* fractal analysis techniques to quantify the scaling properties of the luminance scaling properties of the artworks we indeed found little correlation between *PMF* and their fractal dimensions (0.05). However, it’s important to note that DBC and *SDBC* are just 2 possible approaches to measuring the fractal dimensions of art works, and future research should focus on a more comprehensive study of the relationship between *PMF* and these scaling parameters.

**Fig 19:**
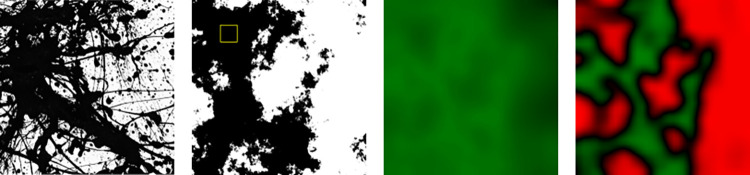
Left: A 63.5 x 63.5cm section of Pollock’s *Number 32*, *1950* (269 x 457.5cm, Kunstsammlung Nordrhein-Westfalen, Germany). Middle-left: A 100 x 100cm computer generated fractal image. Middle-right: the Map of the fractal image. Right: the Map constructed from 10cm tiles (this tile size is shown in the middle left image as a yellow square).

To explore Pollock’s fractal pourings further, we compare them to computer-generated fractal images. Fig 19 demonstrates the visual similarity between the multi-scaled structure of Pollock’s *Number 32* and that of a fractal pattern generated using a midpoint displacement method [75]. The Map of the fractal image is also shown in Fig 19 and is constructed using the multi-scaled tile technique described for the poured paintings in the Methods section. This Map reveals large regions of green that darken in the black and white regions of the fractal, suggesting that the machine is identifying Pollock Signatures mainly at the fractal boundaries between the black and white regions. This effect is confirmed by examining a Map of the fractal image constructed from 10cm tiles. At this scale, it is very clear that the fractal image’s open regions of white generate the red color associated with low Pollock Signatures and that the green color associated with high Pollock Signatures is focused in regions where there are high densities of fractal boundaries. This observation is highly relevant for art theory investigations of Pollock’s work. Whereas boundary lines are traditionally used in illustrations to differentiate the subject from the background, Pollock has been heralded as the artist who re-invented the role of the line for abstract works. In Pollock’s poured works, the line itself is the subject of his work. Fig 19 demonstrates that the fractal character of the lines is responsible for his Signature.

**Fig 20:**
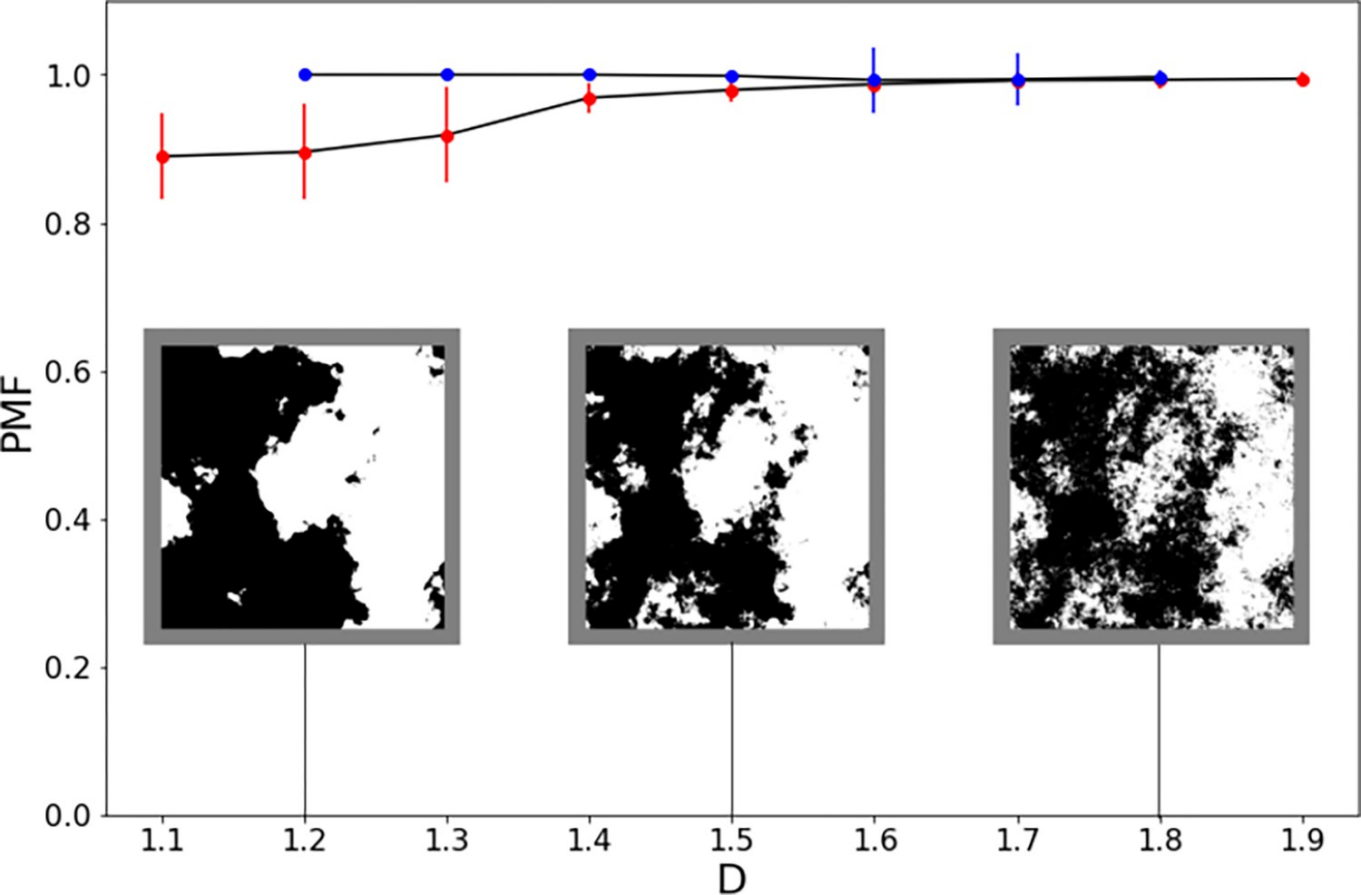
Plots of *PMF* versus fractal dimension *D* for the computer-generated fractals (red) and for Pollocks (blue). The inserts show example fractal images for *D* = 1.2, 1.5, and 1.8.

We examine a total of 63 computer fractals with fractal dimensions *D* spanning from 1.1 to 1.9 in steps of 0.1. The mean *PMF*s are plotted against their *D* values in Fig 20. The high *PMF*s (~ 1) observed for large *D* values fall off gradually but systematically at lower *D*. This can be understood by examining the impact of *D* on the visual appearance of the inserts shown in Fig 20. As described in the Methods section, *D* quantifies the relative contributions of the fine and coarse-scale structures in the fractal mix of the image. Larger *D* values have higher fine scale contributions than the equivalent low *D* fractals, resulting in longer and more complex fractal boundaries between the black and white regions. Considering the 10cm Map shown in Fig 19, this will result in larger Signatures for the high *D* patterns. Fig 20 indicates that Pollock’s poured work is immune to this fall off. The most likely reason for this relates to pattern density. Whereas the computer fractals are set to have 50:50 coverage of the black and white regions, the Pollock paintings typically have much higher densities of painted regions, so cramming even more fractal boundaries into a given region. This maintains their high Signatures down to low *D* values.

**Fig 21:**
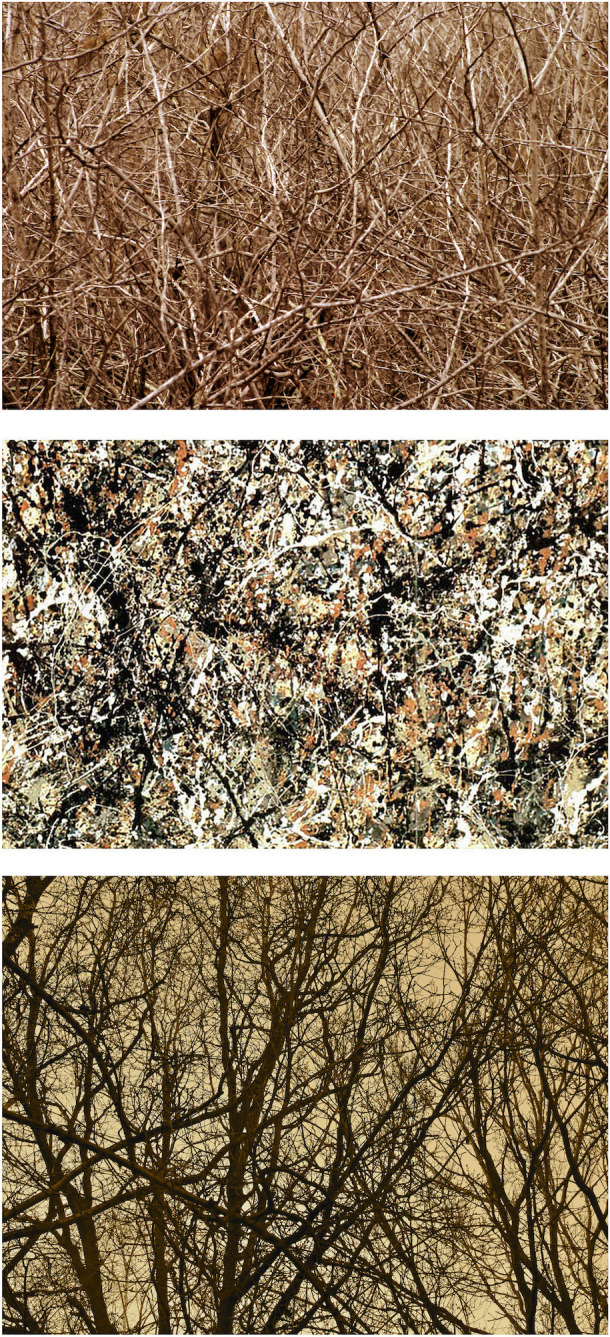
Comparisons of a section of Pollock’s *One*: *Number 31*, *1950* (269.5 x 530.8cm, MoMA, New York) (middle image) to photographs of a thicket (top image) and trees (bottom image).

Given the prevalence of fractals in natural scenery, in Fig 21 we present a preliminary comparison of Pollock’s *One* (1950) with 2 common natural images (a thicket and trees). All 3 images display the multi-scaled structure associated with fractal patterns. Intriguingly, the thicket is quantified by PMF = 0.71, *One* is quantified by *PMF* = 1, while the trees are quantified by *PMF* = 0.52. One possible explanation relates to differences in the fractal boundary density. In particular, the trees feature noticeably larger gaps devoid of the fractal boundaries. However, this result is also a useful reminder that although fractal analysis plays an important role, the machine is more than a simple fractal detector and many other visual characteristics will be in play. Although all 3 images are fractal, the natural images will differ in many subtle ways. A future investigation will perform a systematic study of a comprehensive set of natural images with the aim of using *PMF* to explore the connection between Pollock’s and nature’s fractal patterns.

**Fig 22:**
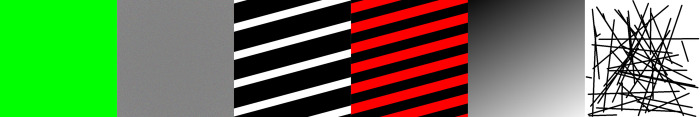
Sample thumbnails of the test patterns. From left to right (single colors, gradient grayscale images, black and white rows (at various angles), colored rows (at various angles), gradient grayscale images (at various angles), and pickup sticks.

Finally, we compare Pollock’s images to a set of standard non-fractal designs to confirm that these are quantified by *PMF* = 0. These patterns encompass solid colors, bars, random pixels, gradients, and the distinctive ‘Pick up sticks’ configuration (which consisted of randomly oriented black lines against a white background). Examples are shown in Fig 22. Our model demonstrates remarkable accuracy in classifying each of the test patterns as being a non-Pollock—with one intriguing exception involving a specific orientation (a 5-degree angle from the horizontal axis) and widths of black and white bars. The vulnerability to a specific line angle is surprising given that Pollock’s all-over style is celebrated for being insensitive to painting orientation. Future research will examine trajectory orientation of Pollock’s work in more detail to determine if there are stand-out orientations of individual features that ‘average out’ when combined into the dense interacting web of the all-over style. Interestingly, we note that the 8 poles within *Blue Poles* are much steeper than the 15-degree condition that challenged our model.

### Image classifications and misclassifications: Example cases

The Pollock Dial shows that all but 2 non-Pollocks lie safely below *PMF* = 0.56, generating our model’s high *MA* for detecting the visual miss-match of non-Pollock works. This is impressive given that our study features a diverse set of 284 poured works created by other artists. This set spans from images taken from Pollock’s studio floor (as noted earlier, this is categorized as a non-Pollock in training because it lacks his compositional approach) to the poured creations of a Monkey.

A number of the non-Pollock poured paintings are by other famous artists. Pollock’s contemporary Max Ernst features in the training (*Young Man Intrigued by the Flight of a Non-Euclidean Fly* from 1942–47) and inference (*The Bewildered Planet* from 1942) sets. Works by Abstract Expressionist contemporaries Hans Hofmann (*Fantasia* from 1943 and *The Wind* from 1944) and Arshile Gorky (*One Year The Milkweed* from 1944) feature in the training set. Artists Marcel Barbeau and Jean-Paul Riopelle from the French Canadian movement Les Automatists, which had a similar artistic mission to the American Abstract Expressionism movement, feature in the training and inference sets. From the 1960s, Niki de Saint Phaelle’s art created by shooting at balloons filled with paint (*Shooting Picture 1 and 2* from 1961) features in the training set, along with Norman Rockwell’s famous parody of Pollock’s work (*The Connoisseur* from 1962). Four of Michael Badwin’s 1980s series (Pollock-inspired poured paintings featuring embedded images) appear in the training set and 2 appear in the Inference set. Sam Francis’s *Untitled* from 1985 appears in the inference set. Eight of Prince Jurgen von Anhalt’s ‘jet art’ (poured paintings created by flinging paint behind a jet engine) appear in the training set and 1 appears in the inference set.

#### Notable ‘imitations’

In terms of well-known imitations, the training set includes 2 paintings created for the Pollock movie by actor Ed Harris, along with 2 paintings by Francis O’Connor, one of the leading Pollock connoisseurs. Forty-two works by the modern-era imitator Mike Bidlo feature in the training and inference sets. Paintings from 2 high-profile disputes are also used, as follows. Eight paintings from the Matter Collection (No.’s 2, 4, 7, 9, 10, 14, 17 and 19) [25] feature in the training set and 1 (No. 3) features in the inference set. Two Knoedler Gallery paintings [3] feature in the training set. Whether used in training or inference, the variety of these poured creations help to deliver the high *MA* of our machine model.

**Fig 23:**
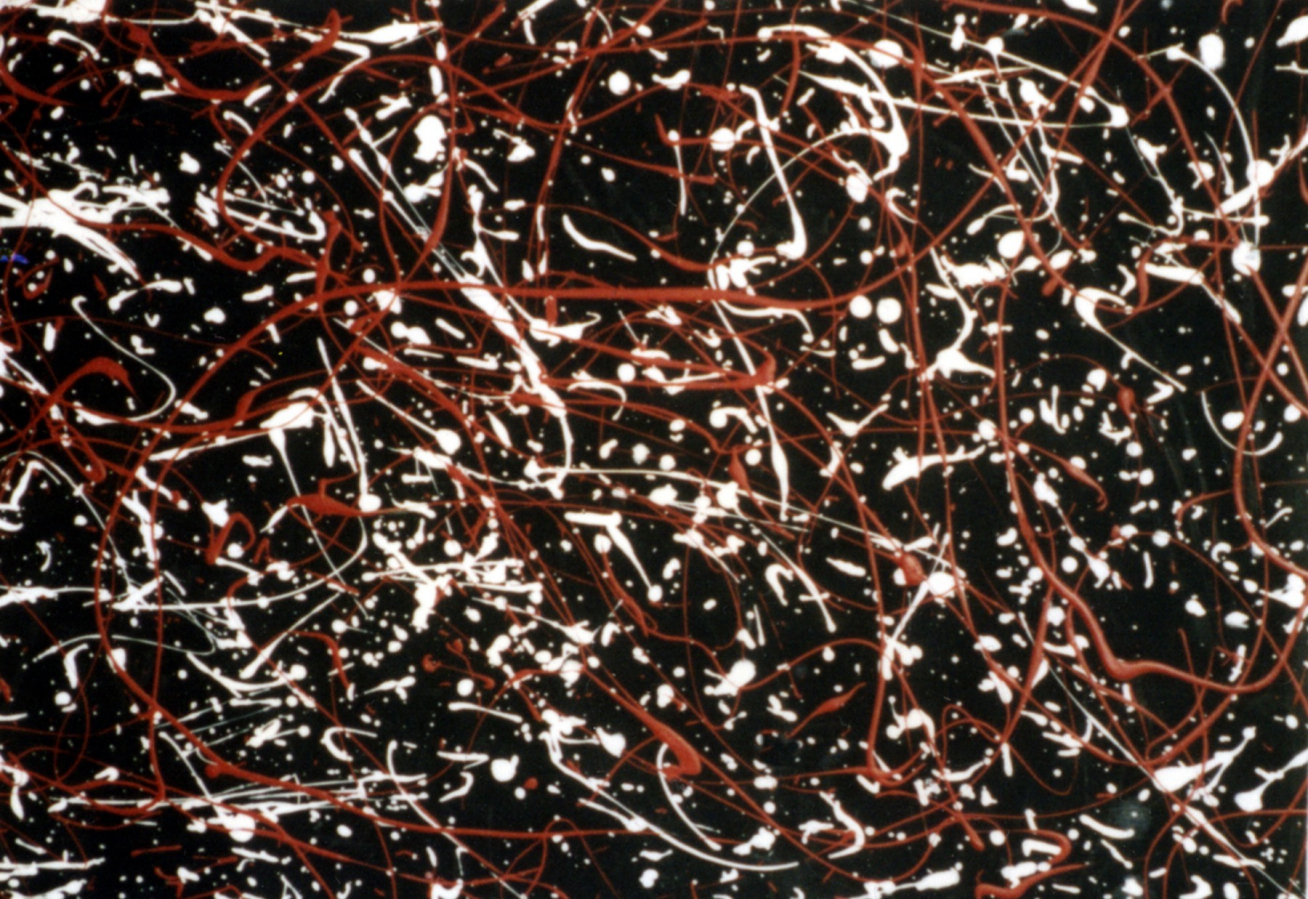
Poured painting generated by the wind machine (*PMF* = 0.11).

The inference set also features an image generated by a wind-driven pendulum built to harness fractal wind gusts to generate Pollock-like fractal patterns (Fig 23) [14]. Despite this intriguing method of capturing nature’s dynamic patterns, our machine is able to classify the resulting painting correctly as a ‘non-Pollock’ (*PMF* = 0.11). Finally, we also use paintings from Dripfests—the experiments aimed at exploring whether children’s poured paintings have more visual similarities than the equivalent adult paintings to Pollocks [17,31]. This hypothesis is based on proposed similarities between the body motions of Pollock and the children. Fig 4 shows example artworks by an adult and a child. Our machine is able to classify both the children’s and adults’ art works correctly as ‘non-Pollocks’. Furthermore, the *PMF*s for the children (mean *PMF* = 0.002 ± 0.002) and adult (mean *PMF* = 0.009 ±0.007) paintings aren’t significantly different between the 2 groups.

#### The Pollockizer

**Fig 24:**
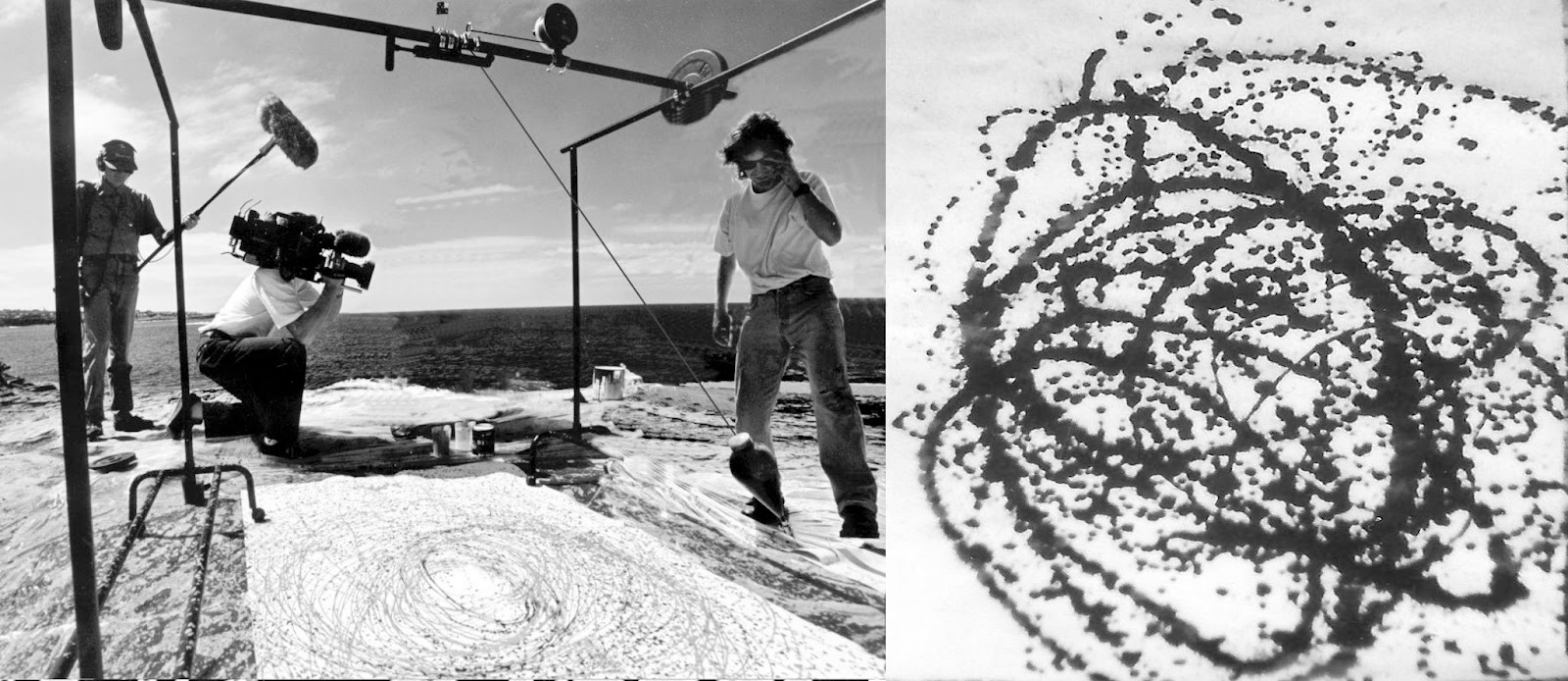
The Pollockizer (left) and a section of the poured painting that is classified as sharing the visual signatures of a Pollock (*PMF* = 0.67) (right).

Our model misclassifies *One* generated by the ‘Pollockizer’—a mechanical device developed to generate Pollock imitations (Fig 24) [35]. This device consists of a container of paint that swings on a string, dripping paint onto a horizontal canvas positioned below. Based on the principle of a chaotic pendulum, the string can be knocked (either mechanically or via magnets) at close to the resonant frequency of the swinging motion. In doing so, fractal patterns are generated in the container’s motion and therefore also in the paint trajectories recorded by the canvas below. Our machine suggests that, by generating a painting with *PMF* = 0.67, the Pollockizer is indeed capable of generating Pollock’s artistic signatures.

#### *AI*-generated art

Given the growing prevalence of machine-generated art (e.g. those generated by DALL-E), a crucial question concerns whether our *AI* technique can detect *AI*-generated Pollock imitations. We test 87 ‘*AI* Pollocks’ and classify them correctly with 100.00% accuracy. These consist of 3 groups of *AI*-generated Pollocks. DALL-E takes a textual description as an input and generates an image that corresponds to that description. We use the textual description “jackson pollock’ and “jackson pollock imitation” to generate images at 1024x1024 pixels and these are tested for 4 different physical dimensions (25x25cm, 50x50cm, 100x100cm, and 200x200cm). *Neural Love* is a web-based tool that uses *AI* to create artistic images, avatars, and realistic portraits. We locate on-line images from *Neural Love* that are proposed to be imitation Pollocks. Their image sizes of 512 x 512 pixels are run through our machine at the same physical dimensions used for the DALL-E paintings. Although an algorithmic approach rather than strictly an *AI* approach, we also use *Pollock Master* images. This is a public github repository that makes Pollock-like images. The images are 5528 × 3572 pixels in resolution and are run through our machine using the same physical dimensions used for the DALL-E paintings [76].

#### Henri Michaux

**Fig 25:**
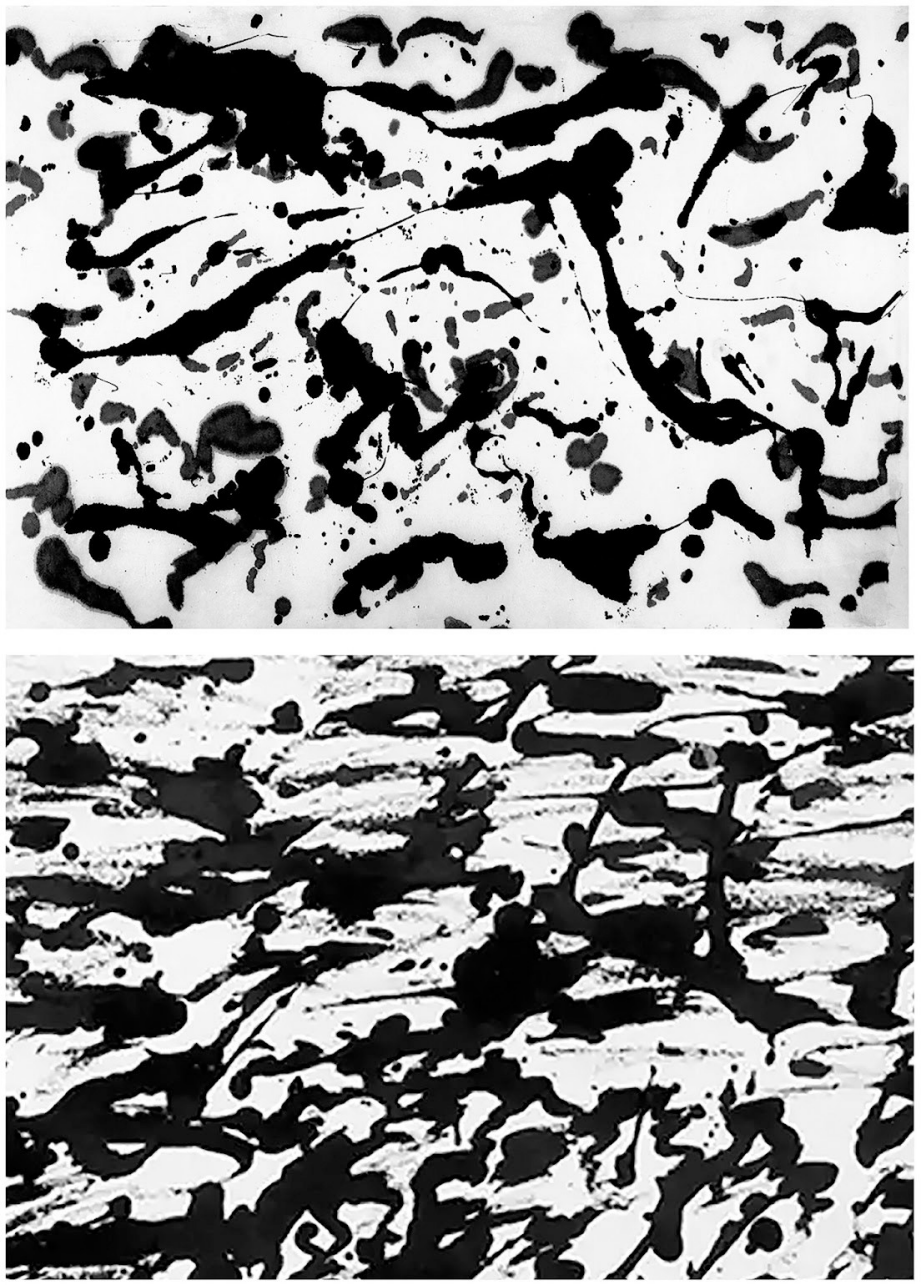
A comparison of sections of Henri Michaux’s *SansTitre 1960–61* (bottom, *PMF* = 0.74) with Pollock’s *Untitled* 1950 (top, *PMF* = 1.00).

As shown in the Pollock Dial, our model misclassifies *Untitled* by Henri Michaux (*PMF* = 0.95). Michaux’s poured works belong to the Tachisme art movement, the French analogue to Pollock’s Abstract Expressionism. Accordingly, we conducted further testing of our model using a data set comprising 9 additional Michaux works [77,78]: 4 works register *PMF* = 0 (indicating a lack of visual resemblance to Pollock’s style), 3 works fall within the range of *PMF* values greater than 0.25 but less than the Threshold, and 2 paintings have a *PMF* exceeding 0.56, suggesting a higher degree of visual similarity to Pollock’s distinctive style (these are *SansTitre 1960–61* and *SansTitre*). This varied outcome of Michaux’s work underscores the complexities involved in distinguishing artistic signatures. The close match of some of his works to Pollock’s Signatures highlights their parallel artistic missions despite their geographic separation. Fig 25 compares a 13.2 x 19.4cm section of Michaux’s *SansTitre 1960–61* (40.2 x 60cm, Galarie Berthet-Aittouares, Paris) with a 40.5 x 59cm section of Pollock’s *Untitled 1951* (63.5 x 99cm, Lee Krasner Collection). Both are generated by pouring ink onto paper. The similarity of these 2 artists’ signatures is intriguing and will be the focus of further art research.

### Future models

We did not augment the image set in our current model. In machine learning, image augmentations are a set of techniques used to artificially increase the diversity of a data set by applying various transformations to the original images. These transformations can include image rotations, flips, scaling, changes in brightness and contrast, and more. The primary goal of image augmentations is to enhance the model’s ability to generalize patterns from the training data to unseen examples. By introducing variations in the data set, the model becomes more robust and less prone to overfitting, which is known as generalization. Image augmentations are particularly valuable in computer vision tasks such as image classification when having a diverse and representative training data set can significantly improve model performance.

We undertake brief investigations of some additional models that utilize training augmentation but a more thorough investigation is needed beyond the scope of our current work. Below, we discuss 2 additional models that we train. Neither model out-performs our current model in *MA* nor by minimizing the *PMF* difference between images of the same painting taken from the 2 Pollock image groups. However, with the appropriate development, we anticipate that a future model with some augmentations could result in a model that is more generalizable than our current one while potentially maintaining our high *MA* performance.

#### Alternate Model 1 (*MA*: 97.7%)

Augmentations: 50% of the images are converted to grayscale, flipped horizontally, flipped vertically, or rotated in steps of 90 degrees. There are several advantages associated with these augmentations: 1) Introducing flips, mirrors, and rotations removes any directional bias from all paintings. This would be helpful if the correct orientation of a painting is not known. 2) Reducing the color influence forces the model to focus on the spatial patterns generated by the pouring process. This equalizes different image sources, some of which are grayscale and others are color. However, these augmentations introduce the following disadvantages which reduce the *MA*: 1) The correct orientations of Pollock paintings are known. This is therefore a real property of his work that we shouldn’t eliminate. Instead, our current model simply rotates a painting of unknown origin and calculates each *PMF* to allow for alternative orientation options. 2) Color can be an important characteristic of Pollock paintings and so reducing the presence of this characteristic diminishes the model’s ability to distinguish.

#### Alternate Model 2 (*MA*: 97.1%)

**Fig 26:**
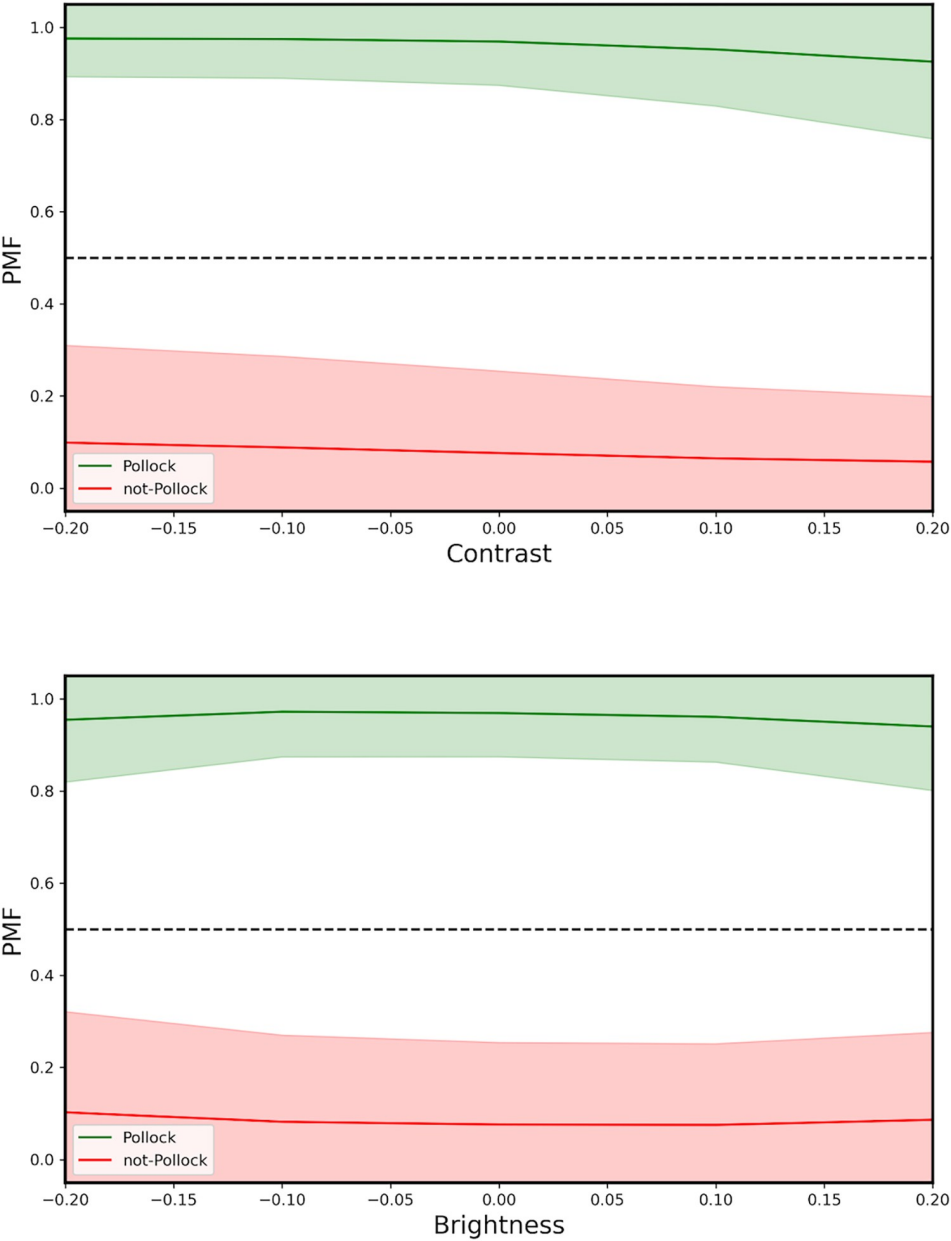
Average *PMF* plotted against the image contrast (top) and image brightness (bottom) for the Pollock (green) and non-Pollock images (red). The broad colored regions correspond to the standard deviations in the data. The horizontal line represents the *PMF* Threshold. In each case, 0 corresponds to the undistorted value on the x axis.

Augmentations: 50% of the images have their brightness and contrast values randomly adjusted between the values of -0.2 to +0.2 (using the Albumentations Python Library). These augmentations increase generalizability to various photographic and lighting conditions. The brightness and contrast plots vs *PMF* become much more stable than those of the current model (see Fig 26). However, these augmentations have the disadvantage that low contrast and brightness could be a real feature of Pollock’s works and this augmentation therefore inappropriately reduces the *MA*.

In summary, augmentations have the potential to improve future models provided any challenges to computational resources are overcome. Lacking augmentations, our current model has a high *MA* and is robust to image variations through careful selection of image parameters. In particular, our model should only be run on paintings of unknown origin that have color images, limited image distortions such as printing artifacts, and limited brightness and contrast distortions.

## Conclusion

We have developed a machine learning strategy that employs a novel image ingestion approach and that leverages the power of transfer learning. Our approach distinguishes between authentic and imitation Pollock poured works with an accuracy of 98.9% despite the limited number of images available for training. Based on this accuracy, we anticipate that our technique will be useful when combined with more traditional approaches to authenticity studies, in particular visual inspections by connoisseurs. For example, returning to the real and imitation *Blue Poles* shown in Fig 1, the real work delivers *PMF* = 1 while the imitation delivers *PMF* = 0.

Our generalization tests show that our machine performs well for artists who use the pouring technique. However, we emphasize that our current model is only applicable to poured paintings. Our *PMF* Threshold is termed a ‘necessary but not sufficient condition’ for image classification tasks. As an example, the high *PMF*s achieved by the computer-generated fractals are useful indicators of the machine’s decision-making process, but they do not challenge the machine’s usefulness as an authenticity tool—because they are not poured works. Further generalization studies will be needed to determine the extent to which our machine can be broadened to other artists and other artistic techniques. It is likely that when trained appropriately our machine will perform well when confronted with the visual complexity generated by other forms of gestural art. As *AI* examinations of art become more widespread, it will be important to understand the limitations of their applicability. For example, techniques based on facial recognition [79] will not be expected to perform equally well when faced with Pollock’s complexity. Authenticity studies will most likely benefit from the employment of diverse machine models.

We have also shown that our *AI* approach can contribute to Pollock studies in ways that move beyond authenticity studies. To counter the black box nature of our approach, we developed Pollock Maps and Pollock Zoom-in Charts to probe the spatial and scaling signatures of Pollocks. When coupled with their quantifying parameters, these novel visual aids provide an interdisciplinary bridge between the machine’s output and traditional art theory investigations of Pollock’s work. In this way, *AI* has the potential to provide a new ‘eye’ on Pollock’s all-over style.

Using these interpretational methods to look into the black box, our results indicate that the machine is examining the scale invariance of the poured patterns in its quest to distinguish between the Pollock masterworks and the imitations. A number of research groups have previously used various forms of fractal analysis to investigate Pollock’s work, and it has been successfully employed in 2 high profile authenticity cases. However, researchers selected their techniques in these previous cases—introducing an element of subjectivity to the process. Here, the machine learned the usefulness of fractal analysis in an objective process beyond human influence. This objective approach will be useful not only for future comparisons of Pollocks with imitations but also with comparisons of Pollocks with natural images. Pollock famously declared “I am nature” to the art world and now science has the tools to confirm this connection between his fractals and those of nature using the machine’s *PMF* values. Intriguingly, recent environment psychology research models the eye as a sophisticated fractal detector [43]. Perhaps Pollock used his fractal eye to spot fractals in nature, the Pollock experts use their fractal eye to spot fractals in Pollock’s work, and now the machine can do the same in a more quantifiable manner.

Finally, allowing *AI* to ‘view’ Pollock’s art represents a major step in art appreciation and represents the latest step in the technological story set in motion by Taylor’s first use of computer analysis of artworks [13]. Writing about Taylor’s computer analysis at the time, MOMA’s chief conservator Jim Coddington declared: “In the visual arts we are at the beginnings of such a field and make no mistake, it is coming.” [29]. In the intervening years, the concept of *AI* has changed from science fiction to science fact and its arrival in the world of art will have many fascinating repercussions.

## Supporting information

S1 Fig Parameter testing. (DOCX)

S1 Table Image categories. (DOCX)

S2 Table Model parameters. (DOCX)

S3 Table Model list. (DOCX)

S4 Table List of PMFs of pollock art works (averaged across the image used for each work). (DOCX)
