# Supplementary material for: Using machine learning to distinguish between authentic and imitation Jackson Pollock poured paintings: A tile-driven approach to computer vision
Source: PLoS One. 2024 Jun 17;19(6):e0302962. doi: 10.1371/journal.pone.0302962 (PMC11182551; doi:10.1371/journal.pone.0302962)
Supplement: S1 Fig — (DOCX) [file pone.0302962.s001.docx]

# **S1 Fig: Parameter Testing**


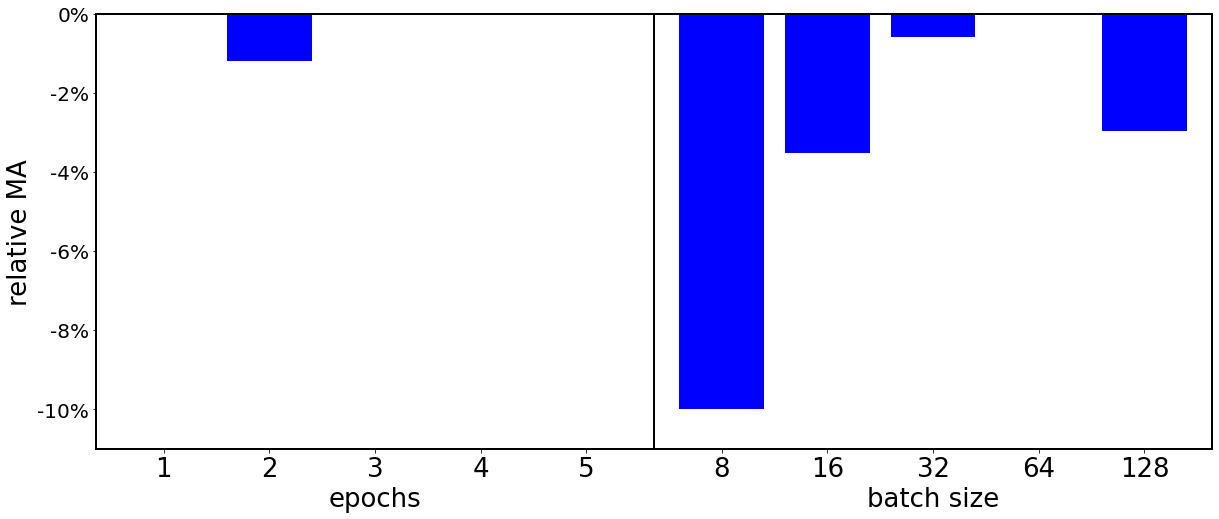


Relative performance across epochs and batch size for Resnet50 with parameters from S2 Table
