# Supplementary material for: Using machine learning to distinguish between authentic and imitation Jackson Pollock poured paintings: A tile-driven approach to computer vision
Source: PLoS One. 2024 Jun 17;19(6):e0302962. doi: 10.1371/journal.pone.0302962 (PMC11182551; doi:10.1371/journal.pone.0302962)
Supplement: S1 Table — (DOCX) [file pone.0302962.s002.docx]

# **S1 Table: Image Categories**

| **Artist** | **Description** | **# of paintings** | **Image Type** | **Source** | **Used in Model Selection** | **Example**  **Image** |
| --- | --- | --- | --- | --- | --- | --- |
| Jackson Pollock | Established Pollock Poured Paintings | 180 | RGB scans of printed grayscale images | *Catalogue Raissonne:*  Ref. [36] | Yes | 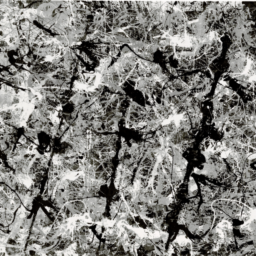 |
| Jackson Pollock | Established Pollock Poured Paintings | 128 | RGB scans of printed color images | Refs. [50-62] | Yes | 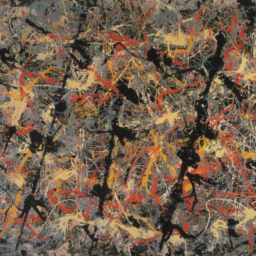 |
| Various (Not Pollock) | Poured paintings known to be not by Pollock | 134 | RGB scans of printed images and transparencies, digital photographs, internet downloadss | The Pollock-Krasner Foundation, Pollock-Krasner Study Center, International Foundation for Art Research, R.P. Taylor, F.V. O’Connor, Internet, private collectors. | Yes | 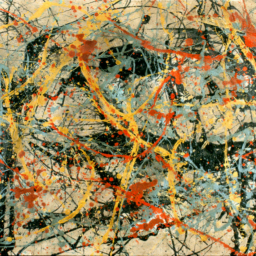 |
| Various (Not Pollock) | Poured paintings generated by adults in the Dripfest | 32 | RGB digital photographs | Photographs from Ref. [17] investigation | Yes | 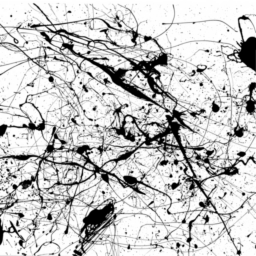 |
| Various (Not Pollock) | Poured paintings generated by children in the Dripfest | 18 | RGB digital photographs | Photographs from Ref. [17]  investigation | Yes | 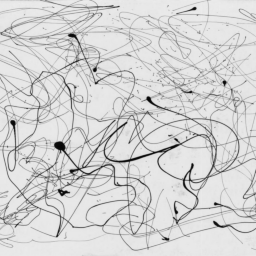 |
| Various (Not Pollock) | Other abstract paintings by famous artists | 115 | RGB scans of printed color images | Ref. [63] | Yes | 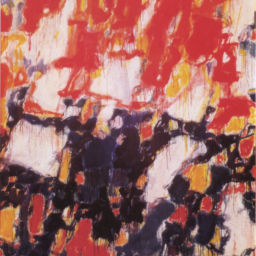 |
| Various (Not Pollock) | Images of poured paintings generated commercially | 100 | RGB electronic downloads | Internet | Yes | 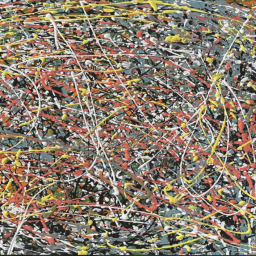 |
| NA | AI generated Images | 87 (346 images) | RGB electronic files | Dall-E, Pollock Master, Neural Love internet toolsls | No | 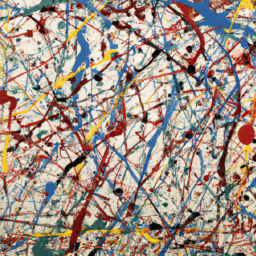 |
| NA | Computer Generated Test images | 6 types (870 images) | RGB electronic file | Generated by Python codecode | No | 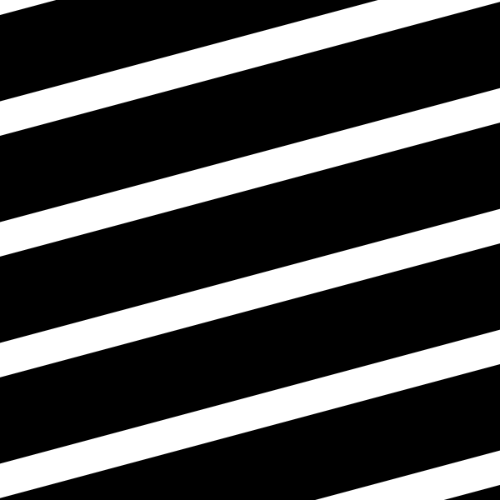 |
| Henri Michaux | Additional Henri Paintings | 9 | RGB scans of printed images, internet downloadsds | Refs. [72,73] and internet | No | 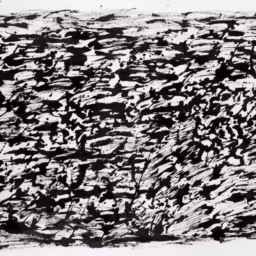 |
| NA | Computer Generated Fractal Images | 63 | RGB electronic files | Refs. [70] | No | 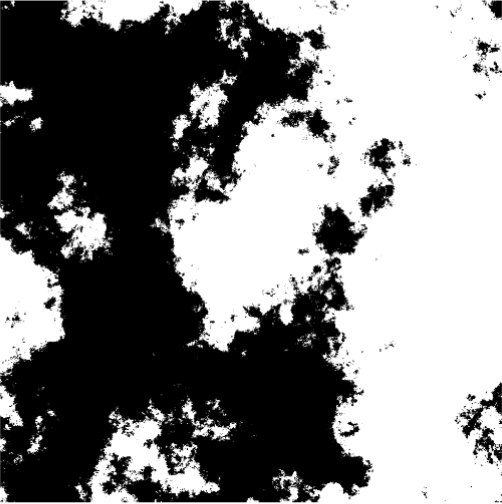 |
