# Supplementary material for: Using machine learning to distinguish between authentic and imitation Jackson Pollock poured paintings: A tile-driven approach to computer vision
Source: PLoS One. 2024 Jun 17;19(6):e0302962. doi: 10.1371/journal.pone.0302962 (PMC11182551; doi:10.1371/journal.pone.0302962)
Supplement: S2 Table — (DOCX) [file pone.0302962.s003.docx]

# **S2 Table: Model Parameters**

| **Parameter** | **Value** | **Reasoning** |
| --- | --- | --- |
| Image Size | 256 x 256 x 3 | Standard input size |
| Batch Size | 64 | Based on total image count, available resources, and  Performance with tuning |
| Epochs | 1 | Performance with tuning |
| Optimizer | Adam | Computationally efficient, optimizes well without much tuning needed |
| Base Learning rate | $10^{-3}$ | Chosen with fastai “lr_find” steepest gradient |
| eps | $10^{-5}$ | Performance without tuning |
| Momentum | 0.9 | Performance without tuning |
| Squared Momentum | 0.99 | Performance without tuning |
| Weight decay | 0.01 | Reduces overfitting |
| Decoupled weight decay | True | Performance without tuning |
