# Supplementary material for: Using machine learning to distinguish between authentic and imitation Jackson Pollock poured paintings: A tile-driven approach to computer vision
Source: PLoS One. 2024 Jun 17;19(6):e0302962. doi: 10.1371/journal.pone.0302962 (PMC11182551; doi:10.1371/journal.pone.0302962)
Supplement: S3 Table — (DOCX) [file pone.0302962.s004.docx]

# **S3 Table: Model List**

| **Arch (short name)** | **Fastaai model name** | **Type** | **Pretrained Database** |
| --- | --- | --- | --- |
| ResNet50 | resnet50 | CNN | ImageNet 1k |
| ResNet34 | resnet34 | CNN | ImageNet 1k |
| ResNet101 | resnet101 | CNN | ImageNet 1k |
| SqueezeNet | squeezenet1_1 | CNN | ImageNet 1k |
| AlexNet | alexnet | CNN | ImageNet 1k |
| **Arch (short name)** | **Hugging Face Hub ID (timm/)** | **Type** | **Pretrained Database** |
| Swin | swinv2_cr_tiny_ns_224.sw_in1k | Vision Transformer | ImageNet 1k |
| DenseNet | densenet121.ra_in1k | CNN | ImageNet 1k |
| PVT | pvt_v2_b5.in1k | Vision Transformer | ImageNet 1k |
| EfficientNet | efficientnet_b5.sw_in12k | CNN | ImageNet 12k |
| MaxVit | maxvit_base_tf_512.in1k | Vision Transformer | ImageNet 1k |
| LeViT | levit_128.fb_dist_in1k | Vision Transformer | ImageNet  1k |
| VOLO | volo_d5_512.sail_in1k | Vision Transformer | ImageNet 1k |
| TinyViT | tiny_vit_21m_512.dist_in22k_ft_in1k | Vision Transformer | ImageNet 22k, fine tuned on ImageNet 1k |
