# Supplementary material for: Using machine learning to distinguish between authentic and imitation Jackson Pollock poured paintings: A tile-driven approach to computer vision
Source: PLoS One. 2024 Jun 17;19(6):e0302962. doi: 10.1371/journal.pone.0302962 (PMC11182551; doi:10.1371/journal.pone.0302962)
Supplement: S4 Table — (DOCX) [file pone.0302962.s005.docx]

# **S4 Table: List of PMFs of Pollock Art Works (averaged across the images used for each work)**

| **catalog** | **title** | **year** | **set** | ***PMF*** | ***SI*** | ***M*** | ***C*** | ***U*** |
| --- | --- | --- | --- | --- | --- | --- | --- | --- |
| 92 | Composition With Pouring 1 | 1943 | train | 1.00 | 1.00 | 9.00 | 1.00 | 1.00 |
| 93 | Water Birds | 1943 | hold_out | 0.78 | 0.67 | 5.00 | 0.90 | 0.95 |
| 94 | Composition With Pouring II | 1943 | train | 1.00 | 1.00 | 5.25 | 1.00 | 1.00 |
| 1088b | Greeting Card | 1943 | valid | 1.00 | 1.00 | 2.00 | 1.00 | 1.00 |
| 130 | Moon Vessel | 1945 | train | 1.00 | 1.00 | 4.00 | 1.00 | 1.00 |
| S36 | Untitled 1946 | 1946 | hold_out | 0.96 | 0.92 | 1.00 | 1.00 | 0.96 |
| 164 | Shimmering Substance | 1946 | train | 1.00 | 1.00 | 6.00 | 1.00 | 1.00 |
| 162 | Eyes In The Heat | 1946 | train | 1.00 | 1.00 | 10.50 | 1.00 | 1.00 |
| S25 | Yellow Collage | 1946 | hold_out | 0.90 | 0.74 | 4.50 | 1.00 | 0.98 |
| 166 | Red Composition | 1946 | train | 1.00 | 0.99 | 4.50 | 1.00 | 1.00 |
| 165 | Free Form | 1946 | hold_out | 0.80 | 0.64 | 3.25 | 1.00 | 0.99 |
| 170 | Composition With Black Pouring | 1947 | train | 1.00 | 1.00 | 2.00 | 1.00 | 1.00 |
| 1019 | Untitled 1947 | 1947 | train | 1.00 | 1.00 | 5.00 | 1.00 | 0.99 |
| 171 | Watery Paths | 1947 | train | 1.00 | 1.00 | 8.50 | 1.00 | 1.00 |
| 172 | Magic Lantern | 1947 | train | 1.00 | 1.00 | 5.00 | 1.00 | 1.00 |
| 174 | The Nest | 1947 | train | 1.00 | 1.00 | 3.00 | 1.00 | 1.00 |
| 176 | Prism | 1947 | train | 1.00 | 1.00 | 4.00 | 1.00 | 1.00 |
| 182 | Shooting Star | 1947 | valid | 1.00 | 1.00 | 6.00 | 1.00 | 1.00 |
| 173 | Enchanted Forest | 1947 | valid | 1.00 | 1.00 | 11.00 | 1.00 | 1.00 |
| 169 | Galaxy | 1947 | train | 1.00 | 1.00 | 8.50 | 1.00 | 1.00 |
| 175 | Reflection Of The Big Dipper | 1947 | train | 1.00 | 1.00 | 9.00 | 1.00 | 1.00 |
| 183 | Phosphorescence | 1947 | train | 1.00 | 1.00 | 6.50 | 1.00 | 1.00 |
| 179 | Alchemy | 1947 | hold_out | 1.00 | 1.00 | 11.00 | 1.00 | 1.00 |
| 180 | Full Fathom Five | 1947 | train | 1.00 | 1.00 | 7.50 | 1.00 | 1.00 |
| 181 | Comet | 1947 | train | 1.00 | 1.00 | 4.25 | 1.00 | 1.00 |
| 184 | Cathedral | 1947 | train | 1.00 | 1.00 | 8.50 | 1.00 | 1.00 |
| 185 | Lucifer | 1947 | train | 1.00 | 1.00 | 10.00 | 1.00 | 1.00 |
| 178 | Vortex | 1947 | train | 0.99 | 0.98 | 4.50 | 1.00 | 0.99 |
| 177 | Sea Change | 1947 | train | 1.00 | 1.00 | 10.50 | 1.00 | 1.00 |
| 206 | Number 18, 1948: Black, Red, Yellow | 1948 | train | 1.00 | 1.00 | 8.50 | 1.00 | 1.00 |
| 203 | Black, White And Gray/Number 11A | 1948 | train | 1.00 | 1.00 | 8.00 | 1.00 | 1.00 |
| 197 | Number 16A, 1948 | 1948 | train | 1.00 | 1.00 | 3.50 | 1.00 | 1.00 |
| 186 | Number 1A, 1948 | 1948 | train | 1.00 | 1.00 | 15.83 | 1.00 | 1.00 |
| 193 | Number 25A, 1948: Yellow Ochre Scro | 1948 | train | 1.00 | 1.00 | 8.50 | 1.00 | 1.00 |
| 190 | Number 19, 1948 | 1948 | valid | 1.00 | 1.00 | 5.50 | 1.00 | 1.00 |
| 216 | White On Black II | 1948 | train | 1.00 | 1.00 | 3.00 | 1.00 | 1.00 |
| 1028 | No Name | 1948 | train | 1.00 | 1.00 | 5.50 | 1.00 | 1.00 |
| 187 | Number 26A, 1948: Black And White | 1948 | train | 1.00 | 1.00 | 11.00 | 1.00 | 1.00 |
| 1032 | Rhythmical Dance | 1948 | train | 1.00 | 1.00 | 6.00 | 1.00 | 1.00 |
| 126 | Figure | 1948 | train | 0.98 | 0.97 | 5.50 | 1.00 | 0.96 |
| 1033 | Untitled | 1948 | train | 1.00 | 1.00 | 5.50 | 1.00 | 1.00 |
| 188 | Number 5, 1948 | 1948 | valid | 1.00 | 1.00 | 12.00 | 1.00 | 1.00 |
| 212 | Square Pouring | 1948 | train | 1.00 | 1.00 | 6.00 | 1.00 | 1.00 |
| 195 | Number 3, 1948 | 1948 | valid | 1.00 | 1.00 | 5.50 | 1.00 | 1.00 |
| 189 | White, Black, Blue And Red On White | 1948 | train | 1.00 | 1.00 | 5.50 | 1.00 | 1.00 |
| 1030 | Cut-Out | 1948 | train | 0.96 | 0.96 | 5.50 | 1.00 | 0.96 |
| 191 | Number 20, 1948 | 1948 | train | 1.00 | 1.00 | 5.00 | 1.00 | 1.00 |
| 192 | Silver Over Black, White, Yellow And Red | 1948 | train | 1.00 | 1.00 | 6.00 | 1.00 | 1.00 |
| 194 | White Cockatoo: Number 24A, 1948 | 1948 | valid | 0.98 | 0.96 | 8.50 | 1.00 | 0.98 |
| 1034 | Shadows: Number 2, 1948 | 1948 | train | 1.00 | 1.00 | 11.00 | 1.00 | 1.00 |
| 198 | Triad | 1948 | train | 0.99 | 0.97 | 5.00 | 1.00 | 0.97 |
| 199 | Number 23, 1948 | 1948 | train | 1.00 | 1.00 | 5.50 | 1.00 | 1.00 |
| 200 | Number 12A, 1948: Yellow, Gray, Black | 1948 | train | 1.00 | 1.00 | 5.50 | 1.00 | 1.00 |
| 201 | Number 22A, 1948 | 1948 | valid | 0.97 | 0.91 | 5.50 | 1.00 | 0.99 |
| 202 | Number 4, 1948: Grey And Red | 1948 | train | 1.00 | 1.00 | 5.50 | 1.00 | 1.00 |
| 204 | Number 14, 1948: Gray | 1948 | train | 1.00 | 1.00 | 5.50 | 1.00 | 1.00 |
| 196 | Number 15, 1948: Red, Gray, White, Yellow | 1948 | train | 1.00 | 1.00 | 5.50 | 1.00 | 1.00 |
| 207 | The Wooden Horse: Number 10A | 1948 | hold_out | 1.00 | 0.99 | 9.00 | 1.00 | 0.99 |
| 208 | Tondo | 1948 | train | 1.00 | 0.99 | 5.50 | 1.00 | 0.97 |
| 209 | Number 6, 1948: Blue, Red, Yellow | 1948 | valid | 1.00 | 1.00 | 5.00 | 1.00 | 1.00 |
| 210 | Number 27A, 1948 | 1948 | valid | 1.00 | 1.00 | 9.00 | 1.00 | 1.00 |
| 211 | Number 17A, 1948 | 1948 | train | 1.00 | 1.00 | 8.50 | 1.00 | 1.00 |
| 215 | Untitled (White On Black I) | 1948 | train | 1.00 | 1.00 | 4.00 | 1.00 | 1.00 |
| 217 | Number 13A, 1948: Arabesque | 1948 | train | 1.00 | 1.00 | 9.00 | 1.00 | 1.00 |
| 205 | Summertime: Number 9A, 1948 | 1948 | train | 1.00 | 1.00 | 8.00 | 1.00 | 1.00 |
| 1031 | Cut-Out Figure | 1948 | train | 1.00 | 1.00 | 5.50 | 1.00 | 1.00 |
| 244 | Number 16, 1949 | 1949 | train | 1.00 | 1.00 | 5.50 | 1.00 | 1.00 |
| 228 | Small Painting | 1949 | valid | 1.00 | 1.00 | 3.00 | 1.00 | 1.00 |
| 227 | Horizontal Composition | 1949 | train | 1.00 | 1.00 | 2.50 | 1.00 | 1.00 |
| 226 | Number 18, 1949 | 1949 | train | 1.00 | 1.00 | 5.50 | 1.00 | 1.00 |
| S5 | Number 21, 1949 | 1949 | hold_out | 1.00 | 1.00 | 4.50 | 1.00 | 1.00 |
| 225 | Number 27, 1949 | 1949 | valid | 1.00 | 1.00 | 3.00 | 1.00 | 1.00 |
| 220 | Number 25, 1949 (triptych) | 1949 | train | 1.00 | 1.00 | 2.50 | 1.00 | 1.00 |
| 221 | Number 29, 1949 (triptych) | 1949 | train | 1.00 | 1.00 | 3.50 | 1.00 | 1.00 |
| 219 | Number 24, 1949 (triptych) | 1949 | train | 1.00 | 1.00 | 2.50 | 1.00 | 1.00 |
| 1035 | No Name | 1949 | train | 1.00 | 1.00 | 5.50 | 1.00 | 1.00 |
| 218 | Number 28, 1949 | 1949 | train | 1.00 | 1.00 | 3.00 | 1.00 | 1.00 |
| 245 | Vertical Painting | 1949 | train | 1.00 | 1.00 | 3.00 | 1.00 | 1.00 |
| 224 | Number 26, 1949 | 1949 | valid | 1.00 | 1.00 | 3.50 | 1.00 | 1.00 |
| 246 | Number 11, 1949 | 1949 | train | 1.00 | 1.00 | 11.00 | 1.00 | 1.00 |
| 251 | Out Of The Web: Number 7, 1949 | 1949 | train | 1.00 | 1.00 | 11.33 | 1.00 | 1.00 |
| 248 | Number 9, 1949 | 1949 | train | 1.00 | 1.00 | 8.50 | 1.00 | 1.00 |
| 241 | Small Composition | 1949 | train | 1.00 | 1.00 | 3.00 | 1.00 | 1.00 |
| 240 | Number 10, 1949 | 1949 | train | 1.00 | 0.99 | 4.50 | 1.00 | 0.99 |
| 239 | Number 8, 1949 | 1949 | valid | 1.00 | 1.00 | 8.50 | 1.00 | 1.00 |
| 238 | Green Silver | 1949 | train | 1.00 | 1.00 | 5.50 | 1.00 | 1.00 |
| 237 | Number 30, 1949/Birds Of Paradise | 1949 | train | 1.00 | 1.00 | 5.50 | 1.00 | 1.00 |
| 236 | Number 15, 1949 | 1949 | train | 1.00 | 1.00 | 5.50 | 1.00 | 1.00 |
| 235 | Number 34, 1949 | 1949 | train | 1.00 | 1.00 | 5.50 | 1.00 | 1.00 |
| 234 | Number 33, 1949 | 1949 | train | 1.00 | 1.00 | 5.50 | 1.00 | 1.00 |
| 233 | Number 12, 1949 | 1949 | train | 1.00 | 1.00 | 5.50 | 1.00 | 1.00 |
| 231 | Number 13, 1949 | 1949 | hold_out | 0.98 | 0.97 | 5.50 | 1.00 | 0.99 |
| 230 | Number 30, (20?) 1949 | 1949 | train | 1.00 | 1.00 | 4.75 | 1.00 | 1.00 |
| 229 | Number 19, 1949 | 1949 | train | 1.00 | 1.00 | 5.50 | 1.00 | 1.00 |
| 223 | Number 23, 1949 | 1949 | train | 1.00 | 1.00 | 3.00 | 1.00 | 1.00 |
| 222 | Number 2, 1949 | 1949 | train | 1.00 | 1.00 | 9.50 | 1.00 | 1.00 |
| 786 | Untitled 1948-49 | 1949 | train | 0.99 | 0.98 | 5.50 | 1.00 | 0.99 |
| 242 | Number 31, 1949 | 1949 | train | 1.00 | 0.99 | 5.50 | 1.00 | 0.98 |
| 247 | Number 6, 1949 | 1949 | train | 1.00 | 1.00 | 11.00 | 1.00 | 1.00 |
| 243 | Number 17, 1949 | 1949 | train | 1.00 | 1.00 | 5.50 | 1.00 | 1.00 |
| 249 | Number 4, 1949 | 1949 | valid | 1.00 | 0.99 | 8.50 | 1.00 | 1.00 |
| 250 | Number 3, 1949: Tiger | 1949 | train | 1.00 | 1.00 | 9.00 | 1.00 | 1.00 |
| 252 | Number 1, 1949 | 1949 | train | 1.00 | 1.00 | 15.50 | 1.00 | 1.00 |
| 255 | Number 22, 1949 | 1949 | train | 0.99 | 0.98 | 5.50 | 1.00 | 1.00 |
| 256 | Number 14, 1949 | 1949 | train | 1.00 | 1.00 | 5.50 | 1.00 | 1.00 |
| 232 | Vertical Painting | 1949 | train | 1.00 | 1.00 | 3.00 | 1.00 | 1.00 |
| 260 | Number 28, 1950 | 1950 | train | 1.00 | 1.00 | 17.00 | 1.00 | 1.00 |
| 288 | Silver And Black Square I | 1950 | train | 1.00 | 1.00 | 5.50 | 1.00 | 1.00 |
| 261 | Number 2, 1950 | 1950 | train | 1.00 | 1.00 | 9.00 | 1.00 | 1.00 |
| 262 | Painting A | 1950 | valid | 1.00 | 1.00 | 5.00 | 1.00 | 1.00 |
| 266 | Number 5, 1950 | 1950 | valid | 1.00 | 1.00 | 9.50 | 1.00 | 1.00 |
| 270 | Number 4, 1950 | 1950 | train | 1.00 | 1.00 | 9.50 | 1.00 | 1.00 |
| 273 | Number 6, 1950/Autumn Landscape | 1950 | train | 1.00 | 1.00 | 8.50 | 1.00 | 1.00 |
| 275 | Number 12, 1950 | 1950 | valid | 1.00 | 1.00 | 5.50 | 1.00 | 1.00 |
| 277 | Number 15, 1950 | 1950 | train | 1.00 | 1.00 | 5.50 | 1.00 | 1.00 |
| 278 | Number 18, 1950 | 1950 | train | 1.00 | 1.00 | 5.50 | 1.00 | 1.00 |
| 279 | Number 14, 1950 | 1950 | train | 1.00 | 1.00 | 5.50 | 1.00 | 1.00 |
| 267 | Number 26, 1950 | 1950 | train | 1.00 | 1.00 | 6.00 | 1.00 | 1.00 |
| 276 | Number 13, 1950 | 1950 | train | 1.00 | 1.00 | 5.50 | 1.00 | 1.00 |
| 1036 | Number 29, 1950 | 1950 | train | 1.00 | 1.00 | 12.00 | 1.00 | 1.00 |
| 292 | Poured Black Shape I | 1950 | valid | 1.00 | 1.00 | 3.00 | 1.00 | 1.00 |
| 298 | Black And White Polyptych | 1950 | valid | 0.97 | 0.89 | 6.00 | 1.00 | 0.97 |
| 297 | Autumn Rhythm: Number 30, 1950 | 1950 | train | 1.00 | 1.00 | 27.00 | 1.00 | 1.00 |
| 290 | Number 10, 1950 | 1950 | train | 1.00 | 1.00 | 9.00 | 1.00 | 1.00 |
| 289 | Silver And Black Square II | 1950 | valid | 1.00 | 0.99 | 5.50 | 1.00 | 0.98 |
| 287 | Number 22, 1950 | 1950 | valid | 1.00 | 0.98 | 5.50 | 1.00 | 0.98 |
| 286 | Number 21, 1950 | 1950 | train | 1.00 | 1.00 | 5.50 | 1.00 | 1.00 |
| 285 | Number 19, 1950 | 1950 | train | 1.00 | 0.99 | 5.50 | 1.00 | 0.99 |
| 283 | One: Number 31, 1950 | 1950 | train | 1.00 | 1.00 | 26.50 | 1.00 | 1.00 |
| 282 | Number 20, 1950 | 1950 | train | 1.00 | 1.00 | 5.25 | 1.00 | 1.00 |
| 280 | Number 16, 1950 | 1950 | train | 1.00 | 1.00 | 5.50 | 1.00 | 0.99 |
| 274 | Number 32, 1950 | 1950 | train | 1.00 | 1.00 | 25.00 | 1.00 | 1.00 |
| 272 | Number 7, 1950 | 1950 | train | 1.00 | 1.00 | 6.00 | 1.00 | 1.00 |
| 271 | Number 27, 1950 | 1950 | train | 1.00 | 1.00 | 12.00 | 1.00 | 1.00 |
| 269 | Number 3, 1950 | 1950 | train | 1.00 | 1.00 | 11.50 | 1.00 | 1.00 |
| 268 | Composition With Red Strokes | 1950 | train | 1.00 | 1.00 | 6.50 | 1.00 | 1.00 |
| 265 | Number 8, 1950 | 1950 | train | 1.00 | 1.00 | 9.50 | 1.00 | 1.00 |
| 264 | Lavender Mist: Number 1, 1950 | 1950 | train | 1.00 | 1.00 | 22.00 | 1.00 | 1.00 |
| 263 | Number 25, 1950 | 1950 | train | 1.00 | 1.00 | 2.50 | 1.00 | 0.99 |
| 259 | Untitled Mural | 1950 | valid | 1.00 | 1.00 | 18.00 | 1.00 | 1.00 |
| 854 | Untitled 1952-56 | 1950 | train | 1.00 | 1.00 | 4.50 | 1.00 | 1.00 |
| 291 | Silver Square | 1950 | train | 1.00 | 1.00 | 5.50 | 1.00 | 1.00 |
| 281 | Green And White Square | 1950 | train | 1.00 | 1.00 | 5.50 | 1.00 | 1.00 |
| 801 | Untitled | 1950 | hold_out | 0.99 | 0.96 | 4.00 | 1.00 | 0.99 |
| 797 | Untitled 1950 | 1950 | train | 1.00 | 1.00 | 2.50 | 1.00 | 0.99 |
| 301 | Red Vertical Composition 5 | 1950 | train | 1.00 | 1.00 | 2.50 | 1.00 | 1.00 |
| 293 | Number 17, 1950/Fireworks | 1950 | valid | 1.00 | 1.00 | 5.50 | 1.00 | 1.00 |
| 294 | Poured Black Shape II | 1950 | valid | 1.00 | 1.00 | 1.00 | 1.00 | 1.00 |
| 295 | Black Over Yellow | 1950 | train | 1.00 | 1.00 | 5.50 | 1.00 | 1.00 |
| 296 | Number 9, 1950 | 1950 | train | 1.00 | 1.00 | 9.00 | 1.00 | 1.00 |
| 299 | Vertical Composition I | 1950 | train | 1.00 | 1.00 | 2.50 | 1.00 | 1.00 |
| 300 | Black And White Triptych | 1950 | train | 1.00 | 1.00 | 5.50 | 1.00 | 1.00 |
| 310 | Silver And Black I | 1950 | train | 1.00 | 1.00 | 2.00 | 1.00 | 1.00 |
| 311 | Silver And Black I | 1950 | valid | 1.00 | 1.00 | 3.50 | 1.00 | 1.00 |
| 312 | Silver And Black Diptych | 1950 | valid | 1.00 | 1.00 | 2.50 | 1.00 | 1.00 |
| S7 | Number 24, 1950 | 1950 | hold_out | 0.95 | 0.87 | 2.50 | 1.00 | 0.92 |
| 802 | Untitled | 1950 | train | 1.00 | 1.00 | 4.00 | 1.00 | 1.00 |
| 808 | Untitled | 1950 | train | 1.00 | 1.00 | 4.00 | 1.00 | 1.00 |
| 792 | Untitled | 1950 | train | 1.00 | 1.00 | 4.50 | 1.00 | 0.99 |
| 284 | Number 11, 1950 | 1950 | valid | 1.00 | 1.00 | 5.50 | 1.00 | 1.00 |
| 315 | Two-sided Painting 1950/1951 | 1951 | train | 1.00 | 1.00 | 6.00 | 1.00 | 1.00 |
| 316 | Number 4, 1951 | 1951 | train | 1.00 | 1.00 | 6.00 | 1.00 | 1.00 |
| 317 | Brown And Silver | 1951 | train | 1.00 | 1.00 | 10.50 | 1.00 | 1.00 |
| 1002 | No Name | 1951 | train | 1.00 | 1.00 | 5.00 | 1.00 | 1.00 |
| 1038 | No Name | 1951 | train | 1.00 | 1.00 | 5.50 | 1.00 | 1.00 |
| 815 | Number 7, 1951 | 1951 | valid | 1.00 | 1.00 | 7.00 | 1.00 | 1.00 |
| 824 | Untitled | 1951 | train | 1.00 | 1.00 | 6.00 | 1.00 | 1.00 |
| 318 | Brown And Silver II, 1951 | 1951 | valid | 0.99 | 0.92 | 10.50 | 1.00 | 0.99 |
| 818 | Number 3, 1951 | 1951 | valid | 1.00 | 1.00 | 6.00 | 1.00 | 1.00 |
| 825 | Untitled | 1951 | train | 1.00 | 1.00 | 6.00 | 1.00 | 1.00 |
| 820 | Untitled | 1951 | hold_out | 1.00 | 0.99 | 6.00 | 1.00 | 0.99 |
| 823 | Untitled | 1951 | train | 1.00 | 1.00 | 6.00 | 1.00 | 1.00 |
| 347 | Composition On Green, Black And Tan | 1951 | train | 1.00 | 1.00 | 5.00 | 1.00 | 1.00 |
| 816 | Number 8, 1951 | 1951 | train | 1.00 | 1.00 | 6.00 | 1.00 | 1.00 |
| 826 | Number 18, 1951 | 1951 | train | 1.00 | 1.00 | 6.00 | 1.00 | 1.00 |
| 346 | Number 28/1951 | 1951 | train | 1.00 | 1.00 | 7.50 | 1.00 | 1.00 |
| 366 | Black Pouring Over Color | 1952 | train | 1.00 | 1.00 | 5.00 | 1.00 | 1.00 |
| 360 | Number 2B, 1952 | 1952 | train | 1.00 | 1.00 | 1.50 | 1.00 | 1.00 |
| 363 | Convergence: Number 10, 1952 | 1952 | train | 1.00 | 1.00 | 22.50 | 1.00 | 1.00 |
| 365 | Yellow Islands | 1952 | train | 1.00 | 1.00 | 14.00 | 1.00 | 1.00 |
| 367 | Blue Poles: Number 11, 1952 | 1952 | hold_out | 0.99 | 0.93 | 21.00 | 1.00 | 1.00 |
| 855 | Untitled | 1952 | valid | 0.97 | 0.92 | 4.50 | 1.00 | 0.98 |
| 364 | Number 12, 1952 | 1952 | valid | 0.96 | 0.75 | 22.00 | 1.00 | 0.99 |
| 358 | Number 1, 1952 | 1952 | train | 1.00 | 1.00 | 6.50 | 1.00 | 1.00 |
| 370 | Greyed Rainbow | 1953 | train | 1.00 | 1.00 | 18.00 | 1.00 | 1.00 |
| 371 | Unformed Figure | 1953 | hold_out | 0.99 | 0.96 | 13.00 | 1.00 | 0.98 |
| 372 | The Deep | 1953 | train | 1.00 | 1.00 | 14.75 | 1.00 | 1.00 |
| 379 | Frieze | 1953 | train | 1.00 | 1.00 | 6.50 | 1.00 | 1.00 |
| 380 | White Light | 1954 | hold_out | 1.00 | 1.00 | 9.50 | 1.00 | 1.00 |
